# Electrolytic Ni-P and Ni-P-Cu Coatings on PCM-Loaded Expanded Graphite for Enhanced Battery Thermal Management with Mechanical Properties

**DOI:** 10.3390/ma18010213

**Published:** 2025-01-06

**Authors:** Onur Güler, Mustafa Yusuf Yazıcı

**Affiliations:** 1 Department of Metallurgical and Materials Engineering, Karadeniz Technical University, 61080 Trabzon, Turkey; 2 Department of Mechanical Engineering, Samsun University, 55420 Samsun, Turkey; myusuf.yazici@samsun.edu.tr

**Keywords:** electrodeposition, Ni-P, Ni-P-Cu, thermal energy storage, phase change material, expanded graphite

## Abstract

This study addresses the thermal management challenge in battery systems by enhancing phase change material composites with Ni-P and Ni-P-Cu coatings on phase change material/expanded graphite structures. Traditional phase change materials are limited by low thermal conductivity and mechanical stability, which restricts their effectiveness in high-demand applications. Unlike previous studies, this work integrates Ni-P and Ni-P-Cu coatings to significantly improve both the thermal conductivity and mechanical strength of phase change material/expanded graphite composites, filling a crucial gap in battery thermal management solutions. The results reveal that Ni-P-Cu-coated phase change material/expanded graphite composites exhibit a superior thermal conductivity of 27.1 W/m·K, significantly outperforming both uncoated and Ni-P-coated counterparts. Mechanical testing showed that the Ni-P-Cu coating provided the highest compressive strength at 39.4 MPa and enhanced tensile strength due to the coating’s highly crystalline structure and smaller grain size. Additionally, the phase-change characteristics of the phase change material/expanded graphite composites, with phase transition temperatures between 38 °C and 43 °C, allowed effective heat absorption, stabilizing battery temperatures under 1.25C and 2.5C discharge rates. Voltage decay analysis indicated that Ni-P and Ni-P-Cu coatings reduced polarization effects, extending operational stability. These findings suggest that Ni-P-Cu-coated phase change material/expanded graphite composites are highly effective in thermal management applications, especially in battery systems where efficient heat dissipation and mechanical durability are critical for performance and safety. This study offers a promising approach to improving energy storage systems for applications such as electric vehicles, grid storage, and portable electronics.

## 1. Introduction

Considering fossil fuels’ economic and environmental impacts, addressing energy needs through electricity requires advancements in energy storage technologies. Lithium-ion batteries (LIBs) have emerged as promising solutions, particularly for meeting the rising demand for alternative energy in electric and hybrid vehicles [1,2,3]. Among energy storage options, LIBs are recognized for their essential qualities: low self-discharge rate, high stability, high power and energy density, and efficient long service life [4,5]. However, during use, especially at high charge or discharge rates and in hotter conditions, LIBs generate significant heat. If this heat is not effectively dissipated or if it accumulates unevenly within the cell, it can drastically reduce battery lifespan and efficiency, even leading to potential fire hazards [6,7]. LIBs can reach an energy density of up to 705 Wh/L and a power density as high as 10,000 W/L. Thermal increase, therefore, is a primary concern affecting LIB performance, efficiency, and longevity, making thermal control systems vital [8]. Extensive research explores device-scale thermal management systems to enhance LIB performance and manage heat in challenging conditions. Thermal management systems aim to stabilize LIB system temperatures and ensure uniform heat dissipation from the battery structure [9,10,11].

Active cooling systems, such as airflow or liquid systems with fans or pumps, rely on external mechanisms. In contrast, passive systems use heat-dissipation fins, heat pipes, or phase change materials (PCMs) to manage heat through phase transitions [12,13,14]. Liquid cooling systems are gaining interest in LIB thermal management due to their high thermal conductivity. However, they require additional components such as channels, valves, fans, and pumps, which increase weight, occupy space, and reduce cooling efficiency by consuming more power. The core of an LIB cell experiences the highest temperatures due to thermal resistance between the core and the cooling medium. Increasing convection alone may worsen temperature gradients, highlighting the need for strategies that remove heat directly from the core for more uniform dissipation [15,16,17,18]. Passive cooling methods utilizing latent heat during phase transitions are increasingly studied. PCMs absorb significant energy during phase shifts, enhancing heat extraction and dissipation in LIB systems. Unlike traditional methods, PCM-based cooling offers stable phase transition temperatures, improved efficiency, and low maintenance requirements, making it a popular choice for passive thermal management [19,20]. Studies show that adding expanded graphite (EG) to paraffin-based PCMs significantly boosts thermal conductivity, making it ideal for LIB cooling. For instance, Xia et al. recently demonstrated that PCM combined with EG outperformed natural air cooling [21]. Similarly, Yang et al. concluded that effective passive thermal management with EG and PCM requires optimizing PCM’s thermal conductivity ratio [22]. Hybrid PCMs reinforced with graphene have also shown enhanced conductivity, resulting in lower LIB temperatures by Aslan et al. [23]. These designs help maintain LIBs within safe operating temperatures (85 °C to 120 °C) [24,25]. Among PCM additives, EG stands out for its high thermal conductivity. Sari and Karaipekli found that adding 10% EG to paraffin increased thermal conductivity by around 272% [26]. EG’s electrical conductivity is also advantageous for LIB applications, as it efficiently conducts heat away from storage devices [27]. Therefore, EG and PCM combinations are actively pursued in LIB thermal management, with significant efforts dedicated to enhancing these materials’ properties [28,29,30]. In a recent study, Yazıcı developed LIB packs with around 92% PCM embedded in a compressed EG structure, showing 33% better heat dissipation than natural air cooling [31]. Accordingly, PCM/EG structures, proven superior to other systems, are chosen as primary materials for LIB thermal control systems in the current study.

Surface treatments and coatings can be used to enable LIB packs to provide cooling and uniform heat dissipation without additional systems. In this context, the literature emphasizes that the increase in connection temperature and heat generation rate can lead to severe device failures and thermal runaway; therefore, excess generated heat must be distributed to the environment via an appropriate heat transfer pathway. Various thermal interface materials (TIMs) are used to enhance thermal performance in heat-generating systems. Since most TIMs are semi-solid, they may face challenges such as pumping under high pressure or drying out in hot conditions, reducing the heat transfer capacity of electronics. Considering these differences, metal-based coatings are an alternative approach to TIMs for heat-dissipating substrates [32,33,34,35]. Nickel-phosphorus (Ni-P) is of particular interest due to its high thermal conductivity [36,37,38]. Ni-P coatings applied to various substrates (copper [39], steel [40], graphite [41]) have been shown to enhance thermal stability and improve thermal management properties due to their high thermal conductivity. Ternary coating methods (Ni-P-Cu [42], Ni-P-Ag [43]) have been developed to further enhance the hardness and thermal properties of Ni-P coatings. In these systems, Cu and Ag particles occupy gaps within the Ni-P matrix, resulting in a denser coating with improved mechanical properties. Adding metals like silver (Ag) and copper (Cu), which possess excellent electrical and thermal conductivity (>380 W/m·K), makes these ternary coatings appeal in thermal management systems. Upon comparing thermal conductivities, silver and copper are noted at 429 W/m·K [44] and 386 W/m·K [45], respectively. Considering the high cost of silver relative to copper, copper is a more attractive option in thermal management systems. Ternary coating systems such as Ni-P-Cu are expected to improve the thermal and mechanical properties of coated substrates by leveraging the high thermal conductivity of copper and the crystalline stability of the Ni-P structure. In a study, Nur Ariffah et al. [46] reported that Ni-P-Ag coatings applied to steel achieved a thermal conductivity of approximately 465 W/m·K. Although studies examine the tribological and structural properties of Ni-P-Cu coatings on substrates, the thermal properties of these coatings have not been extensively studied in the literature. The commonly used methods for applying binary Ni-P and ternary Ni-P-Cu coatings include electroless plating and electroplating. Electroless plating offers several advantages, such as the ability to produce homogeneous coating without requiring an external electric source, making it particularly suitable for complex geometries [47,48]. However, this method is limited by a relatively slow deposition rate (20–25 µm/h) and the complexity of maintaining bath chemistry, which can restrict its applicability in large-scale or high-performance requirements. Electroplating, on the other hand, allows for faster deposition rates and can achieve coating thicknesses exceeding 1 mm through optimized parameters, providing a significant advantage for applications requiring thicker or more robust coatings. Additionally, electroplating is more versatile and cost-effective compared to advanced techniques like physical vapor deposition (PVD) or chemical vapor deposition (CVD), particularly for substrates with simpler geometries. This study highlights the importance of electroplated Ni-P and Ni-P-Cu coatings due to their superior mechanical strength, thermal conductivity, and scalability, offering a promising solution for thermal management applications where other techniques may fall short [49].

Although studies have reported the application of dual Ni-P coatings on various substrates using the electrolysis method, to the best of our knowledge, there has been no research in the literature examining the effects of electrolysis parameters (such as the distance between the anode and cathode and current density) on the properties of triple Ni-P-Cu coatings. Furthermore, in the reported studies on the production and characterization of triple Ni-P-Cu coatings, the copper content is typically introduced either through environmentally harmful and expensive chemicals (e.g., CuSO_4_ as the copper source or hydrazine hydrate as the reducing agent) or by adding Cu powder into the plating solution, which incurs additional costs. Studies focusing on deriving the Ni-P content from the electrolyte/plating solution and the Cu content from the anode plate used in electrolysis are quite rare. Moreover, while the superior mechanical and thermal properties of electrolytic Ni-P and Ni-P-Cu coatings are well-known, their use in battery pack applications is notably absent in the literature. In this context, the proposed project aims to coat PCM/EG thermal control systems/cooling packs with both Ni-P and Ni-P-Cu layers using the electrolysis method. Based on the insights gained from the aforementioned literature, this study was conceived with the idea that improving the thermal and mechanical properties of PCM/EG packs would make them a strong candidate for LIB thermal management systems. The three primary issues observed in PCM/EG packs are identified as follows: low thermal conductivity values, leakage of PCM material into the external environment upon exposure to high temperatures, and the fragile or soft nature of the EG-PCM composite material, which makes it prone to breaking or tearing. To address these challenges, this study focuses on the following question: “Can high thermal conductivity Ni-P-Cu coatings be utilized to produce economical, mass-producible, sustainable, and long-lasting PCM/EG LIB cooling packs?”. In this regard, given the suitability of the electrolysis method for mass production and the ability to obtain coatings with diverse properties (e.g., coating thickness, reduced particle size of Ni-P and Ni-P-Cu nodules deposited on the substrate, surface morphology, and the quality of the substrate-coating interface), by altering electrolysis parameters, the primary goal is to enhance the thermal and mechanical properties of the PCM/EG systems used in LIB cooling systems while keeping costs in mind. The project proposes utilizing scrap copper plates as a source of Cu content, producing Ni-P-Cu-coated EG/PCM LIB packs via an optimized electrolytic process. These packs are expected to exhibit higher thermal conductivity and greater thermal energy storage capability, providing a significant contribution to affordable, mass-producible, and environmentally friendly thermal control systems for LIB thermal management technology applications. This study will investigate the impact of Ni-P-Cu coatings on mechanical properties, which is expected to attract significant attention from researchers in the field of PCM/EG LIB packs for the first time in the literature. The ability to provide a solution to the search for high-performance thermal management systems that combine complex functionalities (e.g., heat storage and heat transfer) with superior mechanical properties underscores the originality of this study.

## 2. Materials and Methods

### 2.1. Materials

The materials used in this study were sourced from Sigma-Aldrich, Munich, Germany and included expandable graphite, PCM, and chemicals for the electrolyte solution of Ni-P and Ni-P-Cu coatings. The expandable graphite was purchased from Sigma-Aldrich, Munich, Germany. EG has a purity of >98%, a particle size of about 500 µm, and a density of approximately 700 mL/g. It is thermally expanded at 1000 °C, increasing its volume up to 150 times its original size and achieving over 90% porosity to facilitate effective liquid absorption. The PCM type was Rubitherm RT42, obtained from Rubitherm GmbH (Berlin, Germany), which was selected for its high latent heat of fusion (approx. 180 kJ/kg) and melting point around 42 °C. This PCM was chosen for its chemical and thermal stability and compatibility with EG in composite applications for thermal management systems. Nickel sulfate (NiSO_4_) had a purity of 99.9%, providing Ni ions necessary for the electroplating solution. Sodium hypophosphite (NaH_2_PO_2_) had a 99.9% purity, serving as a reducing agent for Ni-P deposition. Tri-sodium sulfate (Na_3_PO_4_) also had a 99.9% purity; this compound was included in the electrolyte to stabilize the Ni-P and Ni-P-Cu coatings. Ammonium sulfate ((NH4)_2_SO_4_) with a purity of 99.9% was added to the electrolyte solution to improve the uniformity of the coating. A 99.9% pure nickel plate was used as an anode for the Ni-P coating process. For Ni-P-Cu coatings, a scrap copper anode with 99.5% purity was used to introduce Cu ions into the electrolyte solution. All chemicals were obtained in high-purity form from Sigma-Aldrich to ensure the consistency and quality of the electroplating solutions used in this study.

### 2.2. Fabrication of PCM/EG Packs

**Figure A1:**
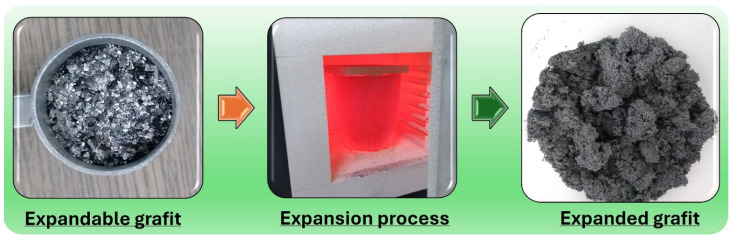
Production of EG particles by expansion of expandable graphite particles.

The flaky graphite particles, obtained as expandable graphite to produce EG particles that form the matrix structure of the PCM/EG composite, were placed in a silicon carbide (SiC) crucible within a muffle furnace heated to 1000 °C and held for 60 s. After this period, the furnace was allowed to cool, yielding EG particles formed by the expansion of the flaky expandable graphite particles within the SiC crucible. The process of obtaining EG particles from flaky expandable graphite particles is shown in Figure A1.

**Figure A2:**
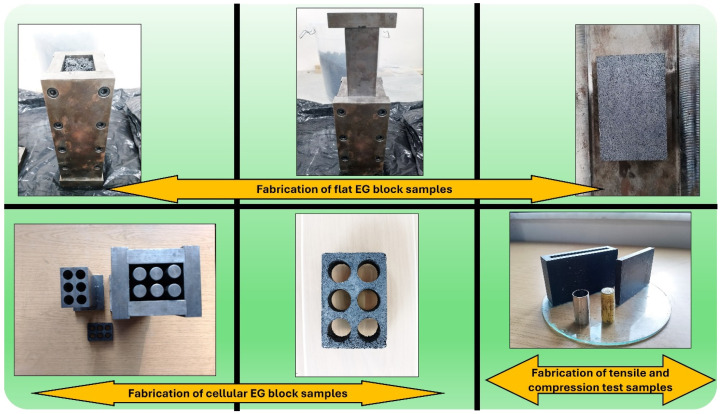
The mold and fabrication of different types of block or bulk samples.

Following the production of expandable graphite particles, to produce EG matrix blocks, the expandable graphite particles were shaped within molds, including a rectangular section mold, a rectangular section mold with cylindrical cores for battery cells, and molds for tensile and compression test specimens (Figure A2). The EG matrix integrated materials produced for these specimens, which were filled into the molds to achieve a density of 0.5 g/cm^3^, a value determined through trial experiments. A density lower than 0.5 g/cm^3^ caused the integrated EG structure to disintegrate upon removal from the mold while applying a higher density resulted in a reduction in the amount of PCM absorbed due to insufficient porosity in later stages. During pressing, a pressure of 20 MPa was applied to shape the samples. This process was designed to ensure that the EG matrix could be removed from the mold without disintegration and to maximize PCM impregnation, thus enhancing heat storage properties. Accordingly, to produce a rectangular section, flat block samples with a volume of 75 × 52 × 20 mm were used to achieve a density of 0.75 g/cm^3^ (75 g/L), and 5.85 g of EG particles were used as exhibited in Figure A2. In total, 3.56 g of EG particles were used to produce rectangular section cellular block samples with six 18 mm-diameter cell spaces assembled with LIB for performing the thermoregulation tests. To produce square section tensile samples with dimensions of 100 × 10 × 10 mm, 0.75 g of EG particles were used as shown in Figure A2. Lastly, for the production of cylindrical section compression samples with a diameter of 10 mm and a height of 20 mm, 0.12 g of EG particles were used as seen in Figure A2. The specified amounts of EG particles were pressed at 20 MPa within the respective molds, preparing the porous EG matrix block samples for PCM impregnation. The samples required for characterization tests were obtained by cutting and separating these specimens after producing the flat block samples.

**Figure A3:**
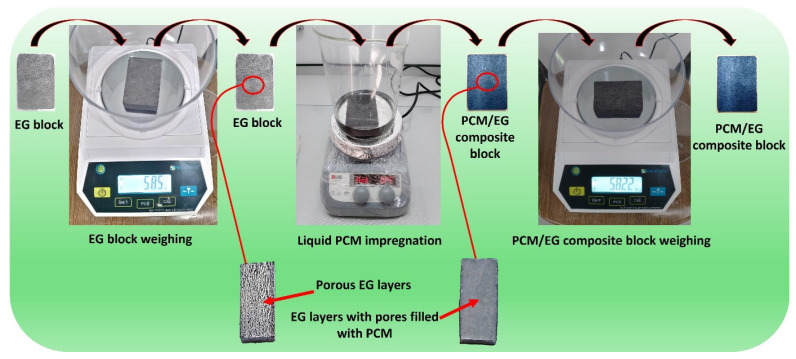
PCM impregnation into the EG blocks.

The production of PCM/EG composite blocks was achieved by immersing the pre-pressed EG blocks in a liquid PCM bath after weighing them. The PCM was impregnated into the EG matrix through surface forces and capillarity. Accordingly, the impregnation process continued for 4 h until the EG matrix blocks reached full saturation with PCM. The PCM was first fully melted in a glass beaker on a heating plate at 70–80 °C, and this temperature was maintained while the prepared EG blocks were immersed in the liquid PCM. After the PCM impregnation, the PCM/EG blocks were weighed using a precision balance. The impregnation process of PCM/EG composites is shown in Figure A3. Due to the high porosity of EG, the EG blocks became fully submerged in PCM as the PCM was absorbed. To demonstrate the densification of the structure by filling the PCM into the EG pores, macro images of cross-sections were taken from small pieces cut from the pressed blocks before and after the PCM impregnation process, as shown in Figure A3. EG blocks without PCM weighed approximately 5.85 g, while after PCM impregnation, their weight increased to about 58.22 g. This indicates that the structure contains approximately 89.95% PCM.

### 2.3. Ni-P and Ni-P-Cu Electrodeposition of PCM/EG Packs

After obtaining the PCM/EG composite packs as described in the previous section, they were left to rest for one day, and these samples were processed for electrolytic Ni-P and Ni-P-Cu coatings. The electrolysis parameters applied for all coating processes are provided in Table 1. In electrolytic coatings, the anode–cathode distance and current density are among the most critical parameters influencing the coating quality and properties. The anode–cathode distance directly affects the uniformity of the electric field and ion distribution in the electrolyte, thereby determining the deposition rate and the homogeneity of the coating [50]. The current density is equally critical as it governs the nucleation and growth of the coating [51]. These parameters were optimized to balance deposition rate, coating homogeneity, and desired mechanical and thermal properties for the current study.

**Figure A4:**
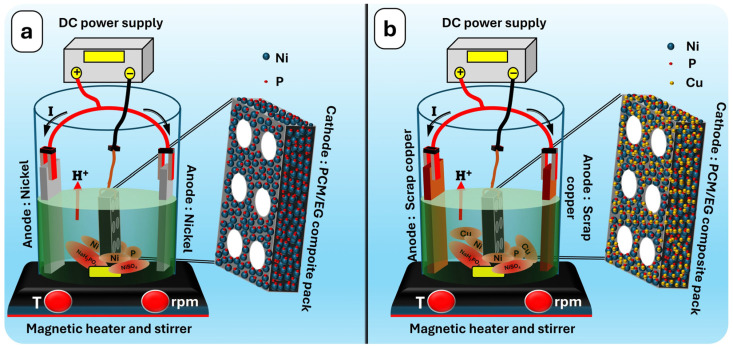
Electrolytic (**a**) Ni-P and (**b**) Ni-P-Cu coating process of the PCM/EG composite packs.

Before the electrolytic coating processes, PCM/EG matrix samples underwent a surface conductivity treatment to enhance electrical conductivity, increase the reduction rate per hour, and improve electrochemical compatibility. In this context, all samples were processed at 40 °C for 5 min in a solution containing 0.2 g/L palladium chloride (PdCl_2_·2H_2_O) and 2 g/L HCl, then rinsed with pure water. The PCM/EG packs were connected as cathodes in the electrolyte, with nickel plates used as anodes for Ni-P coatings and scrap copper plates for Ni-P-Cu coatings. A 0.05 M nickel sulfate (NiSO_4_) solution was used, while 0.05 M sodium hypophosphite (NaH_2_PO_2_) served. Additionally, 0.05 M trisodium citrate was added, and 0.05 M ammonium sulfate was included. The electrolyte solution temperature was set to 30 °C and the stirring speed to 600 rpm. The electrolytic coating processes lasted 10 h. For Ni-P-Cu coating processes, the electrolyte composition was set identical to that used in the Ni-P coating step. The only difference in the electrolytic Ni-P-Cu coating process was the use of scrap copper (99.5% purity) as the anode instead of nickel. The electrolytic Ni-P and Ni-P-Cu coating of the obtained PCM/EG composite packs was carried out as shown in detail in the image provided in Figure A4.

It is known that phosphorus cannot be precipitated alone; rather, it can only be co-deposited along with iron group metals. Such precipitation is classified as induced electroplating. The following mechanisms are shown to explain this process. The three reactions below occur simultaneously and independently. Hypophosphite used as the phosphorus source, participates in reduction reactions along with Ni^2+^ and H^+^ ions. From this, it is understood that the release of H_2_ gas indicates the progression of the coating process. In Ni-P-Cu coatings, Cu is directly reduced onto the cathode from the anodic scrap copper. During these reactions, which occur independently and simultaneously, the reduced Cu ions settle among the Ni-P nodules, as shown in Equations (1)–(4).

H_2_PO^2−^ + 2H^+^ + e^−^ → P + 2H_2_O (1)

Ni^2+^ + 2e^−^ → Ni (2)

2H^+^ + 2e^−^ → H_2_ (3)

Cu^2+^ + 2e^−^ → Cu (4)

### 2.4. Characterization

After the electrolytic Ni-P and Ni-P-Cu coating processes, small samples were cut from the rectangular cross-sectional PCM/EG blocks for microstructure and phase analysis. PCM/EG composites, prepared to their sizes and geometries for tensile, compression, and thermal performance analyses, were individually subjected to the aforementioned coating processes. The powder morphology of the EG particles, the microstructure of the EG block structure obtained from pressed EG particles, the microstructure of the PCM-impregnated EG block structure, and the coating morphology and microstructure after the application of Ni-P and Ni-P-Cu coatings were examined using scanning electron microscopy (SEM, Zeiss Evo LS10, (Carl Zeiss AG, Oberkochen, Germany)). Additionally, energy-dispersive spectroscopy (EDS) mapping was used to observe the distribution of elements within the coating structure. The phase analyses of the EG particles, the bulk EG structure obtained by pressing these particles, the PCM, the PCM-impregnated EG structure, and the coatings after Ni-P and Ni-P-Cu applications were determined using X-ray diffraction (XRD, PANalytical X-Pert3 Powder (Malvern Panalytical Ltd., Almelo, The Netherlands)). In particular, the crystalline size and crystallinity degrees of the obtained coatings were determined to examine their effect on different coating types.

To investigate the effect of Ni-P and Ni-P-Cu coatings on the strength of the PCM/EG matrix composite LIB packs, tensile and compression tests were conducted at room temperature. Tensile tests on the packs were performed on a sample cut in the direction parallel to the compression of the EG block. Square-sectioned samples were cut to the required dimensions and fixed vertically to ensure uniaxial loading. A universal Instron testing device was used to obtain stress–strain diagrams, with the tensile head speed set to 1 mm/min. For compression tests, cylindrical samples of 20 mm length and 10 mm diameter were subjected to uniaxial compressive loading using a universal Instron testing machine, with the head speed set to 1 mm/min. For each sample group, three tensile and three compression tests were conducted. The stress–strain curves were generated by averaging the instantaneous values obtained from each test at corresponding strain points. This approach ensures that the final stress–strain curves represent the mean behavior of the material while minimizing the impact of individual test variability.

Meanwhile, differential scanning calorimetry (DSC, Hitachi 7020 (Hitachi High-Tech Corporation, Tokyo, Japan)), thermogravimetric analysis (TGA, Perkin Elmer TGA 4000 (PerkinElmer, Inc., Waltham, MA, USA)), and thermal conductivity analysis were used to determine the thermophysical properties of the materials. In this scope, the heat capacities, phase transition temperatures, and enthalpies of the materials were determined, and the effect of PCM addition was revealed using DSC analysis. The thermal program of the device was set, with heating and cooling rates selected between 5 and 20 °C/min. Samples were subjected to a controlled temperature program in an atmosphere of nitrogen and argon alongside a reference material. TGA was used to determine the thermal stability and decomposition behavior of the materials. The temperature program of the device was set, with a heating rate between 10 and 20 °C/min. The analysis temperature was set to vary from room temperature to 700 °C in a controlled nitrogen atmosphere. For DSC and TGA analyses, each sample was tested three times to ensure the reliability of the results. The final curves and data points were obtained by averaging all measurements from the three tests. This approach minimizes variability and provides a representative thermal behavior of the materials.

In this study, the thermal conductivity of all materials shaped into blocks was measured using a Linseis THB Analyzer (Linseis Messgeräte GmbH, Selb, Germany). This device operates based on the Transient Hot Bridge (THB) method, which ensures precise evaluation of thermal properties. By utilizing a thin film sensor that acts as both a heater and a temperature sensor, the device provides accurate and reliable thermal conductivity measurements, making it suitable for the wide range of materials studied in this work. Thermal conductivity measurements were conducted three times for each sample group to ensure accuracy and reliability. The final values presented in the graphs represent the averages of these tests, and error bars were included to indicate the standard deviation, reflecting the variability in the measurements.

### 2.5. Thermal Management Tests

**Figure A5:**
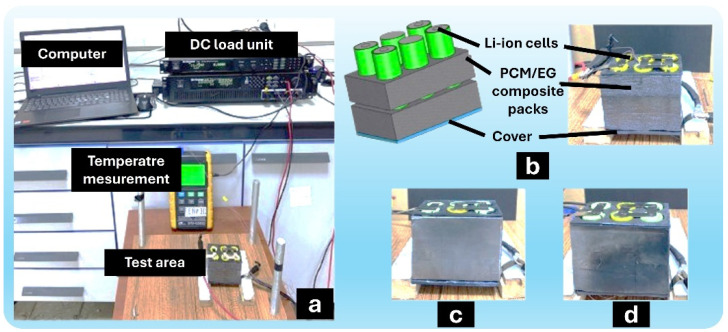
Thermal management test setup images; (**a**) test area, (**b**) assembly of the packs with LBs and placement of batteries in uncoated packs, (**c**) Ni-P-coated packs, and (**d**) Ni-P-Cu-coated packs.

A schematic diagram of the test setup used to determine the effect of Ni-P and Ni-P-Cu coatings on the thermal management performance of PCM/EG packs is shown in Figure A5. Performance tests were conducted in a conditioned chamber/incubator (Nucleon brand) at ambient temperature (T = 25 °C) and discharge current values (8.1 A, 10.2 A). The charging and discharging of the battery pack were performed using a BK PRECISION power supply (9115 DC) (BK Precision Corporation, Yorba Linda, CA, USA) and a DC Electronic Load Unit (8514B) (BK Precision Corporation, Yorba Linda, CA, USA), respectively. Temperature measurements were recorded with a Keithley (Integra Series 2701) (Keithley Instruments, Cleveland, OH, USA) data acquisition system and a PCE-1200 temperature measurement device (PCE Instruments, Meschede, Germany). The test area consists of two main components: the PCM/EG composite pack and the LIB cell (Figure A5a). The test area has main dimensions of 74 × 51 × 60 mm (width × length × height), allowing for the packaging of six Li-ion batteries (Panasonic/Sanyo NCR 18650GA, 3.6 V, 3500 mAh) (Panasonic Corporation, Osaka, Japan) in a 3s2p configuration (Figure A5b). This 3s2p configuration results in a battery pack voltage of 11.7 Volts and a capacity of 6700 mAh. The cells were connected using nickel strip wire. The battery pack is supported at the top and bottom by 3D-printed support parts (5 mm thick, dark blue color). Measurements were taken for uncoated PCM/EG (Figure A5b), Ni-P-coated PCM/EG (Figure A5c), and Ni-P-Cu-coated PCM/EG composite packs (Figure A5d).

**Figure A6:**
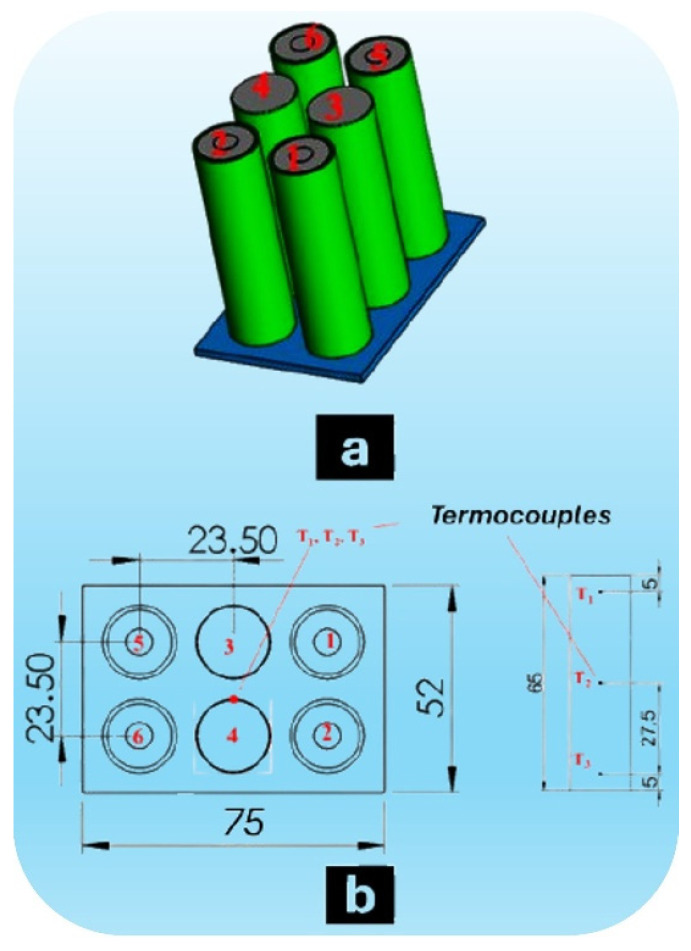
Illustration of thermocouple placement (**a**) locations with (**b**) dimensions on the LIBs.

In the thermal performance evaluation of the LIB packs, temperature measurements were taken from a total of six local points on the battery surface, representing the entire battery pack. Temperature measurements were conducted using T-type thermocouple pairs. Schematic images showing the designated cell and thermocouple placements are presented in Figure A6. For the selected cell, a total of six temperature measurement points were identified—three on the inner and three on the outer surfaces (bottom, middle, and top). T1, T2, and T3 represent the thermocouples attached to the outer surface of the respective cell, while T4, T5, and T6 represent the three thermocouples on the inner surface of cell number 1.

For the thermal management tests, T-s, V-I-s, and V-Ah-Wh graphs were generated using the average data obtained from three independent tests conducted for each sample group. This approach ensures that the final graphs represent the mean performance of the samples, minimizing variability and providing reliable and consistent results.

## 3. Results

### 3.1. Particle Size Distribution and Morphology

**Figure 1:**
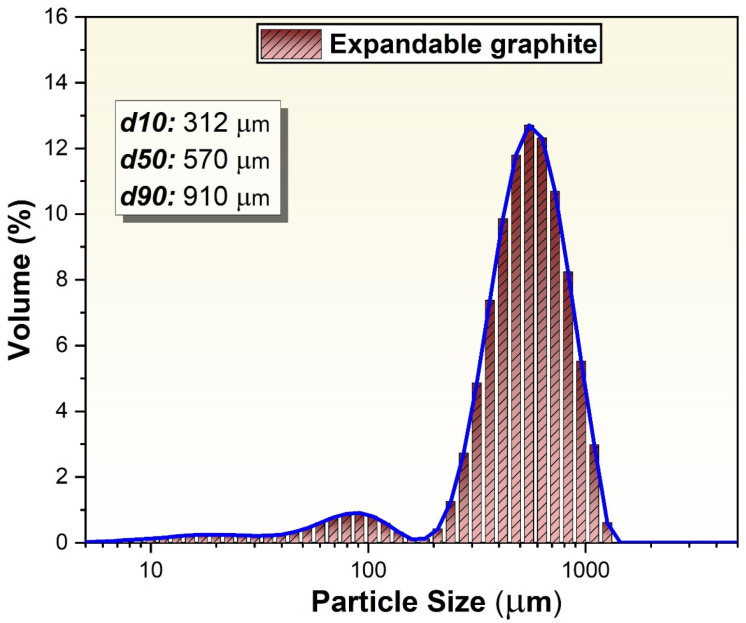
Particle size distribution graph of the expandable graphite particles.

Figure 1 demonstrates the particle size distribution of expandable graphite particles used as initial materials that will be transformed into EG particles in the study. The particle size distribution graph for expandable graphite provides detailed insight into particle size range and distribution. This graph shows how the particle sizes are distributed in terms of volume percentage, with a significant peak around 1000 µm, indicating that most of the particles are concentrated at this size. From the graph, the d50 value, which represents the median particle size, is approximately 570 µm. This means that 50% of the particles are smaller than this size, and 50% are larger, providing a central measure of particle size within the distribution.

To interpret d10 and d90 values from the graph, the d10 value represents the particle size below which 10% of the sample’s particles are found. Based on the graph, the d10 value is approximately 300 µm. This indicates that 10% of the particles are smaller than 300 µm, providing insight into the finer portion of the particle size distribution. d90 value represents the particle size below which 90% of the sample’s particles fall. From the graph, the d90 value is around 900 µm, meaning that 90% of the particles are smaller than 900 µm. This value is useful for understanding the upper size range of the particle distribution, giving an idea of the larger particles present in the sample. The peak around 1000 µm indicates that a high-volume percentage of the sample’s particles are concentrated around this size range, signifying that most of the material is composed of particles close to this diameter. The graph shows a distribution of particle sizes, where each size range contributes a certain percentage to the total sample volume. In the production of EG with a heterogeneous particle size distribution of initial expandable graphite particles, there are several potential positive effects, particularly when the EG particles are pressed. A mixture of different particle sizes allows smaller particles to fill the voids between larger particles, leading to a higher packing density. This can enhance the structural integrity and mechanical strength of the pressed EG material, making it more stable and robust. The presence of both fine and coarse particles increases the surface contact area between particles during pressing. This improves bonding between particles, which is beneficial for creating a dense and cohesive structure in the final product. This phenomenon has been reported in the literature by various researchers [52,53].

**Figure 2:**
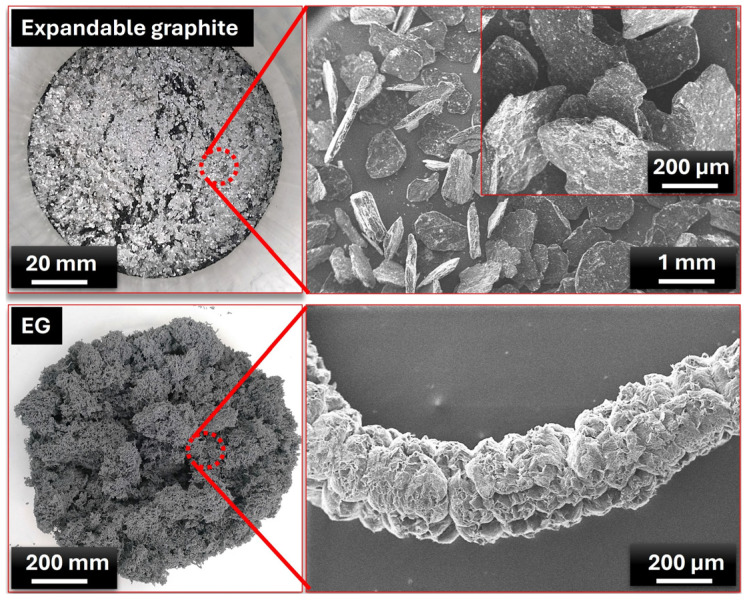
SEM images of the initial expandable graphite and obtained EG particles.

Figure 2 shows the morphological structure of expandable graphite and EG particles, revealing significant insights into their physical transformations and properties relevant to composite applications. The expandable graphite in its initial state can be seen in the upper section of Figure 2. The left-side image displays a macro-scale photograph with a scale of 20 mm, showing the shiny flake-like appearance typical of natural graphite. When magnified on the right, with a scale of 1 mm, the graphite particles exhibit a layered flake morphology, a characteristic feature of expandable graphite. These flakes are closely packed and appear dense and compact, which is crucial as the layered structure allows for significant expansion when subjected to high temperatures. The lower section of the figure illustrates the expanded state of expandable graphite after thermal treatment. The left-side image shows a macro view of EG, where the material exhibits a much larger volume, and a more porous sponge-like structure compared to the compact pre-expanded form. The scale here is 200 mm, emphasizing the large expansion effect achieved. Upon magnification (right image, 200 µm scale), the EG reveals a unique cellular structure, with individual graphite particles that have puffed up and fused into a network of interconnected layers. This porous interlayered structure enhances the material’s surface area and thermal insulation properties, making it highly suitable for thermal management applications. Consequently, the images depict the transformation of expandable graphite into EG, demonstrating the drastic increase in volume and porosity due to thermal treatment. The expanded form’s porous structure is advantageous in applications where thermal conductivity and heat dissipation are essential, as the structure allows for efficient heat transfer while maintaining a lightweight and adaptable framework. These morphological characteristics support the effectiveness of EG in composite materials, especially for PCM composites in thermal energy storage, where the expanded structure can accommodate and support the PCM within its network, as stated in the literature [54,55].

### 3.2. Microstructure of the Bulk EG and PCM/EG Composite Packs

**Figure 3:**
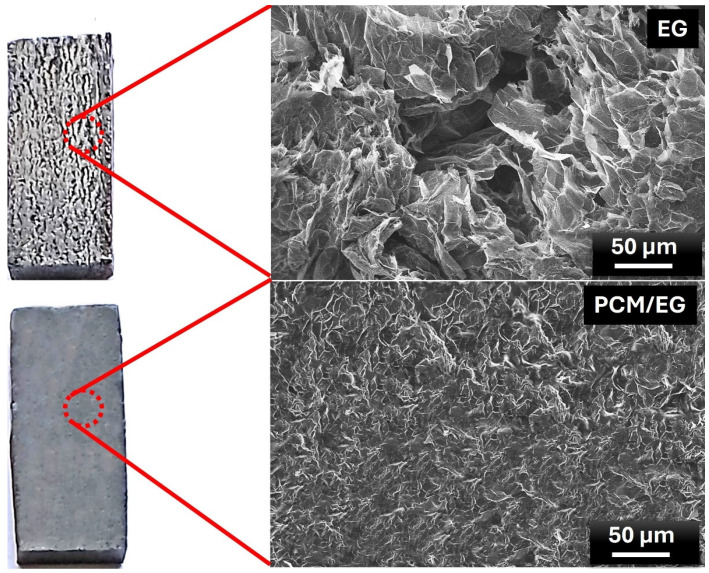
SEM images of the microstructure of the bulk EG and PCM/EG composite packs.

Figure 3 exhibits the macro view and microstructure of EG blocks and PCM/EG composite packs. These results provide valuable insights into the impact of PCM infiltration on the microstructure of the EG matrix. The upper portion of Figure 3 displays a cross-section of a pure EG block without any infiltrated phase change material. On the left, a macro view shows the block’s structural form, while the right image provides a magnified SEM view (50 µm scale). The magnified image reveals a highly porous and layered morphology, which is characteristic of expanded graphite. This structure features thin wrinkled graphite layers with abundant open spaces, creating a network of interconnected pores. This porous morphology is characterized by interconnected voids, allowing gases or liquids to move through the structure. The pore size and distribution are influenced by factors such as heating rate and intercalant concentration. Due to the expanded layers and wrinkled structure, EG possesses a large surface area, enhancing its ability to adsorb liquids or other materials. This property makes EG an ideal candidate for composite applications where the infiltration of PCMs is desired, as often highlighted in the literature [28,56,57,58]. Such morphology enhances thermal conductivity due to the extended graphite surfaces, making expanded graphite a common choice for thermal management applications. The lower portion of Figure 3 illustrates the structural changes after PCM infiltration into the EG block, forming a PCM/EG composite. On the left, the macro view shows a relatively smooth surface compared to the pure EG block, suggesting that the paraffin has penetrated and filled the porous network. The SEM image on the right (50 µm scale) confirms this, showing a denser structure with fewer visible voids. The previously open EG layers appear to be more compact and are covered by PCM, resulting in a less porous and more cohesive structure. This transformation indicates that the PCM effectively fills the EG’s pores, reducing its porosity and potentially enhancing its thermal management properties. The literature suggests that the addition of a PCM in EG structures not only stabilizes the matrix but also enhances the heat storage capacity of the composite [59,60]. When heated, the PCM absorbs heat by melting, while the EG facilitates rapid heat distribution throughout the composite. Upon cooling, the PCM solidifies, releasing stored heat and providing consistent thermal regulation [61].

### 3.3. Thermal Stability and Phase Transition Analysis of the EG, PCM, and PCM/EG Composite Packs

**Figure 4:**
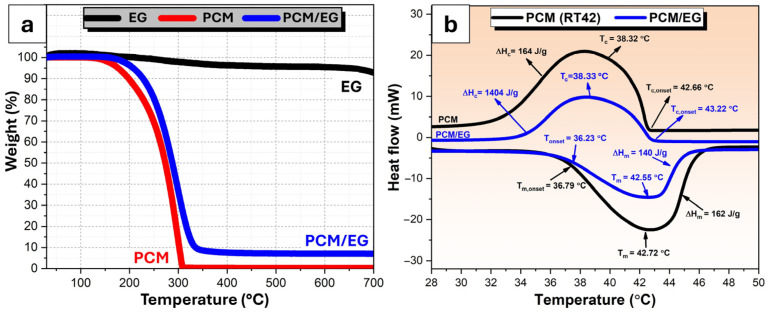
The curves of (**a**) TGA and (**b**) DSC of the EG, PCM, and PCM/EG composite packs.

Figure 4 presents the curves illustrating the TGA and DSC results. In Figure 4a, the TGA results display the weight loss profiles of three samples: pure expandable graphite, pure PCM, and a PCM/EG composite, across a temperature range from room temperature to 700 °C. The weight loss behavior provides insight into the thermal stability and decomposition stages of each material. The TGA curve of EG shows exceptional thermal stability, with a negligible weight loss up to approximately 700 °C. This stability indicates that EG undergoes minimal thermal decomposition or volatilization within this temperature range, making it an excellent structural component in high-temperature applications. The weight loss is primarily due to the decomposition and release of residual oxygen-containing functional groups or intercalated substances, such as sulfuric acid or nitric acid, used during the initial preparation or exfoliation process. This results in a gradual reduction in weight as these components decompose and volatilize under increasing temperatures [62,63]. The pure PCM sample exhibits a significant weight loss starting around 150 °C and continuing until approximately 350 °C, where the weight loss reaches a plateau. This indicates that PCM undergoes thermal degradation within this range, which may involve the evaporation or decomposition of the material’s organic components. The final residual weight is close to zero, indicating that PCM is almost entirely decomposed at high temperatures. The PCM/EG composite demonstrates a weight loss profile that lies between pure PCM and EG. The composite starts to lose weight around the same temperature as pure PCM (approximately 150 °C) but shows a slower rate of decomposition. By 350 °C, the PCM/EG composite retains more weight than pure PCM, suggesting that the presence of EG improves the thermal stability of PCM. This could be due to the protective effect of EG, which may limit the volatilization of the PCM components by creating a physical barrier. This behavior highlights the synergistic effect of the EG on enhancing the composite’s thermal stability as stated by Zhang et al. [64]. Additionally, for the PCM/EG composite, the residual mass was approximately 9%, indicating that almost all PCM was thermally decomposed, leaving behind only the EG fraction. This observation aligns well with the theoretical composition of the composite, which is approximately 90% PCM and 10% EG by weight. The close agreement between the theoretical and experimental results validates the uniform impregnation of PCM within the EG matrix and supports the reliability of the composite preparation method.

In Figure 4b, the DSC results are presented for the PCM and PCM/EG composite, illustrating the melting and crystallization transitions along with their respective enthalpies. The DSC curve of pure PCM (black curve) shows distinct endothermic and exothermic peaks corresponding to melting and crystallization, respectively. During the melting process, the onset melting temperature (T*_m_*_,*onset*_) is observed at approximately 36.79 °C, with a peak melting temperature (T*_m_*) of 42.72 °C as can be seen in Table 2. The enthalpy of melting (Δ*H_m_*) for pure PCM is 162 J/g. For crystallization, the onset temperature (T*_c_*_,*onset*_) is approximately 38.32 °C, with a peak crystallization temperature (T*_c_*) of 42.55 °C and an enthalpy (Δ*H_c_*) of 164 J/g. These values indicate a consistent phase change behavior with significant energy storage capacity, which is crucial for thermal energy storage applications. The DSC curve of the PCM/EG composite also shows melting and crystallization transitions but with slightly altered thermal characteristics compared to pure PCM. The onset melting temperature (T*_m_*_,*onset*_) for the composite is 36.23 °C, with a peak melting temperature (T*_m_*) of 42.55 °C and a reduced enthalpy of melting (Δ*H_m_*) at 140 J/g. For crystallization, the onset temperature (T*_c_*_,*onset*_) is around 42.66 °C, with a peak crystallization temperature (T*_c_*) of 43.22 °C and an enthalpy (Δ*H_c_*) of 1404 J/g. The reduction in melting enthalpy for the PCM/EG composite suggests that the addition of EG decreases the energy storage capacity, possibly due to the dilution effect, where EG occupies volume within the composite, thereby reducing the proportion of active PCM material. However, the enhanced crystallization enthalpy and shifted crystallization temperatures indicate improved crystallization kinetics, which could lead to more efficient heat release during the solidification phase. The combined TGA and DSC results provide a comprehensive understanding of the thermal behavior and stability of the PCM/EG composite compared to pure PCM and EG. The TGA results reveal that EG imparts enhanced thermal stability to PCM, which can be attributed to its high decomposition temperature and protective structure. This enhancement is likely due to the barrier effect of EG, which inhibits the volatilization of PCM components during decomposition, thereby improving the composite’s overall thermal resilience [65,66].

The DSC results further highlight the impact of EG on the thermal properties of PCM. While the melting enthalpy is reduced in the PCM/EG composite, the crystallization process appears to be more efficient, as evidenced by the higher enthalpy value. This effect can be explained by the nucleating role of EG, which may facilitate the crystallization of PCM by providing additional sites for crystal growth. This phenomenon is advantageous in thermal energy storage applications, where a more rapid and efficient heat release during the solidification phase can improve the performance of the material in cyclic heating and cooling applications. In addition, the DSC results allow calculating the PCM content in the PCM/EG composite in percentage terms by comparing the enthalpies of pure PCM and the PCM/EG composite. The melting enthalpy of pure PCM is 162 J/g, while the melting enthalpy of the PCM/EG composite is 140 J/g. The PCM loading percentage in the composite can be estimated by dividing the enthalpy of the PCM/EG composite (140 J/g) by the enthalpy of pure PCM (162 J/g) and then multiplying by 100. This yields approximately 86.4%, indicating that around 86.4% of the composite is PCM, with the remaining 13.6% being EG. This calculation provides insight into the proportion of PCM impregnated into the EG structure in the composite. Similar calculations are frequently used in the literature [67,68]. In conclusion, the slight deviation in the measured enthalpy value compared to the theoretical prediction of approximately 90% PCM and 10% EG can be attributed to factors such as interactions between PCM and EG, restricted phase change within the porous structure, or experimental conditions; the results remain remarkably close. This strong agreement between theoretical and experimental values underscores the reliability of the sample preparation method and the uniformity of PCM distribution within the EG matrix, further validating the composite’s design and performance.

### 3.4. XRD Results of the EG, PCM, and PCM/EG Composite Packs

**Figure 5:**
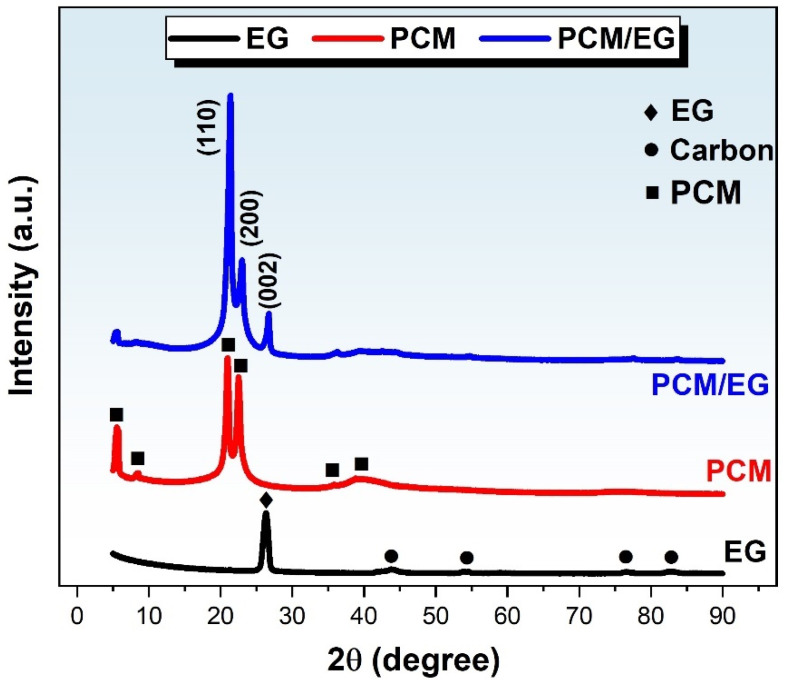
XRD patterns of the EG, PCM, and PCM/EG composites.

Based on the provided XRD patterns shown in Figure 5, the EG sample displays a prominent peak corresponding to the (002) plane at around 26.38°, which is typical for graphitic structures and confirms the presence of graphite layers. The PCM material shows two characteristic peaks at approximately 21.32° and 23.02°, corresponding to the (001) and (200) planes, respectively. These peaks represent the crystalline structure inherent to PCM. The PCM/EG composite exhibits three diffraction peaks that are essentially a combination of the peaks observed in the EG and PCM patterns. This overlap indicates that the composite structure retains the individual crystalline characteristics of both EG and PCM without forming a new crystalline phase. No new diffraction peaks are observed in the PCM/EG composite, suggesting that no novel crystalline structure has formed in the composite. This implies that the composite is an integration of EG and PCM rather than a new distinct material with its crystal structure. All peaks in the PCM/EG composite pattern show a reduction in intensity compared to the individual EG and PCM samples. This decrease in intensity suggests an interaction between the EG and PCM phases, leading to a reduction in the crystallinity or alignment of the individual components within the composite. The reduction in peak intensity may be due to partial disorder or a disturbance in the crystalline structure because of the physical mixing or bonding between EG and PCM during composite formation. The absence of new peaks and the diminished intensity of the original peaks indicate that the PCM/EG composite is structurally a combination of EG and PCM rather than a chemically altered or restructured material. This aligns with the interpretation that the composite material’s properties arise from the combination of EG’s graphitic structure and PCM’s crystalline features. The composite appears to have a homogeneous structure with coherent interactions between EG and PCM, as evidenced by the consistency of the peak reduction. These findings suggest that the composite exhibits a balanced integration of the individual properties of EG and PCM, likely enhancing the material’s mechanical or thermal performance due to the synergy between the two components. The observed peaks for EG and PCM align well with those reported in previous studies [69,70]. This similarity supports the validity of the structural characteristics observed in this study and confirms that the PCM/EG composite follows known structural behaviors for these materials. In conclusion, the XRD analysis of the PCM/EG composite suggests a successful integration of PCM and EG without the formation of a new crystalline structure. The reduction in peak intensities reflects the interaction between the two components, indicating that the composite possesses a homogeneous structure with properties derived from both PCM and EG. This homogeneous integration points to potential enhancements in composite functionality while preserving the distinct structural traits of each component.

### 3.5. Morphology of Ni-P and Ni-P-Cu Coating Layers of PCM/EG Composite Packs

**Figure 6:**
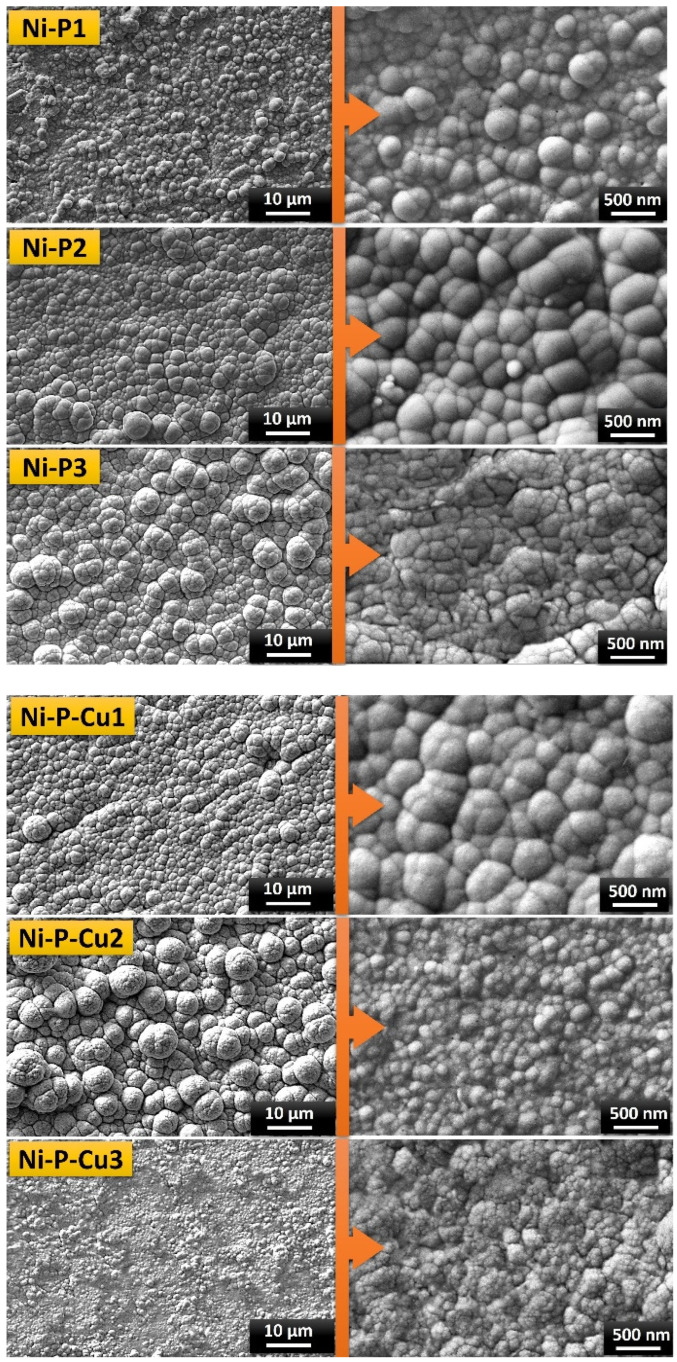
Morphology of the Ni-P and Ni-P-Cu coatings obtained with different coating parameters.

Figure 6 displays the surface morphologies of PCM/EG composite packs coated with Ni-P and Ni-P-Cu under various electrolytic parameters, as shown in Table 1 in the experimental section. The samples, labeled as Ni-P1, Ni-P2, and Ni-P3 for Ni-P coatings and Ni-P-Cu1, Ni-P-Cu2, and Ni-P-Cu3 for Ni-P-Cu coatings, were produced with varying anode–cathode distances and current densities, which impact coating characteristics such as grain size, coating nodules diameters, surface roughness, and overall coating uniformity. The SEM images for Ni-P1 show a relatively smooth and compact coating morphology with small densely packed grains. The lower current density and short anode–cathode distance likely contribute to this compact morphology. Lower current densities tend to promote the formation of finer grains due to the slower deposition rate, which allows atoms to arrange more uniformly, leading to a denser structure. The Ni-P2 coating exhibits a more granular surface with larger grain clusters compared to Ni-P1. Increasing the current density to 2 A/dm^2^ accelerates the deposition rate, which can lead to larger grain growth as atoms have less time to arrange into compact structures. The increased anode–cathode distance may also contribute to the more pronounced grain size by slightly altering the ion flow dynamics and enhancing the mass transfer effects. The Ni-P3 sample presents the most pronounced granular structure, with larger and more distinct grains distributed across the surface. The higher current density (3 A/dm^2^) and increased anode–cathode distance promote rapid nucleation, but the atoms have even less time to arrange into a compact structure, resulting in a rougher surface with more significant grain growth. These conditions facilitate dendritic growth and rougher surfaces due to higher deposition rates and the increased distance affecting ion concentration gradients.

The Ni-P-Cu1 coating exhibits a mixed morphology with both Ni-P and Cu particles evident on the surface. The addition of Cu into the electrolyte tends to modify the deposition process, as copper ions have different deposition kinetics. At a lower current density (1 A/dm^2^) and shorter anode–cathode distance, the Cu may co-deposit with Ni-P in a more controlled manner, leading to a finer smoother surface with the mixed metal distribution. This results in a more uniform coating with copper’s catalytic influence promoting finer grain structures. In the Ni-P-Cu2 sample, the surface morphology shows larger grains and increased surface roughness compared to Ni-P-Cu1. The increased current density (2 A/dm^2^) and moderate distance between electrodes promote faster deposition rates, leading to the formation of larger coarser grains. The Cu in the electrolyte likely induces some stress within the coating as the ions deposit at a faster rate, resulting in more pronounced grain boundaries and increased surface roughness. The Ni-P-Cu3 coating has the roughest and most irregular morphology among the Ni-P-Cu samples. With a current density of 3 A/dm^2^ and the greatest anode–cathode distance, the deposition process is highly accelerated, and the co-deposition of Ni and Cu creates a complex, rough surface. The increased deposition rate can lead to non-uniform coverage and increased surface roughness, which is likely due to rapid nucleation and reduced mobility of atoms on the substrate, preventing the formation of a smooth layer. Additionally, at this high deposition rate, the copper may precipitate in localized regions, further contributing to the irregular surface morphology. In the Ni-P-Cu2 sample, the surface morphology shows larger grains and increased surface roughness compared to Ni-P-Cu1. The increased current density (2 A/dm^2^) and moderate distance between electrodes promote faster deposition rates, leading to the formation of larger coarser grains. The Cu in the electrolyte likely induces some stress within the coating as the ions deposit at a faster rate, resulting in more pronounced grain boundaries and increased surface roughness. The Ni-P-Cu3 coating has the roughest and most irregular morphology among the Ni-P-Cu samples. With a current density of 3 A/dm^2^ and the greatest anode–cathode distance, the deposition process is highly accelerated, and the co-deposition of Ni and Cu creates a complex rough surface. The increased deposition rate can lead to non-uniform coverage and increased surface roughness, which is likely due to rapid nucleation and reduced mobility of atoms on the substrate, preventing the formation of a smooth layer. Additionally, at this high deposition rate, the copper may precipitate in localized regions, further contributing to the irregular surface morphology.

For the Ni-P and Ni-P-Cu coatings on PCM/EG composite packs, the effects of current density and anode–cathode distance on the resulting surface morphology are crucial. For the Ni-P-Cu coatings, higher current densities combined with increased anode–cathode distances appear to favor smaller grain formation. This phenomenon can be attributed to the reduction behavior of copper, where the crystallinity and reduction rate of Cu are influenced by both the deposition conditions and the presence of Ni-P. As the current density increases, the reduction rate of Cu accelerates; however, by also increasing the anode–cathode distance, ion diffusion is balanced in a way that promotes smaller more uniformly distributed grains. This controlled reduction process for Cu enhances the overall structural uniformity within the Ni-P matrix. When the anode–cathode distance is minimized while maintaining a high current density, there is a tendency for inhomogeneity in the deposition process. This is because a smaller distance combined with a high current density can lead to a localized and uneven reduction in ions, which disrupts the uniform nucleation and growth of Cu crystals. Such conditions can result in uneven coating morphologies and lower structural integrity in Ni-P-Cu coatings due to inconsistent reduction and distribution of copper within the matrix. In contrast, for Ni-P coatings, achieving a uniform and efficient reduction process is challenging at high current densities and larger anode–cathode distances. In these conditions, the reduction of Ni-P may become insufficient due to the increased ion path length, leading to inconsistent deposition and larger grain formations. Lower current densities with reduced anode–cathode distances provide better control over the reduction process, enabling finer grain formation in Ni-P coatings. However, for Ni-P-Cu coatings, copper’s selective reduction further influences the deposition morphology. Increasing the Cu content within the electrolyte could enhance the crystalline structure by facilitating the preferential reduction of Cu at high current densities, which contributes to increased crystallinity and grain refinement in the Ni-P-Cu composite structure. The goal of the Ni-P-Cu coatings is to maximize the crystalline nature of the composite, which enhances the electrical conductivity of the PCM/EG composite packs. Higher electrical conductivity is advantageous for applications where thermal management and efficient heat transfer are desired. Consequently, higher current densities combined with increased anode–cathode distances, which allow for greater Cu reduction and deposition, result in coatings with improved electrical properties due to enhanced copper crystallinity. Therefore, lower current densities and shorter anode–cathode distances are insufficient for achieving the desired Cu content and crystalline structure in Ni-P-Cu coatings, as they limit Cu’s reduction rate and distribution. On the other hand, a higher current density with a larger anode–cathode distance is beneficial for Ni-P-Cu coatings, as it promotes a more homogeneous reduction and distribution of copper. This balance ultimately leads to a more crystalline structure, contributing to enhanced electrical conductivity in the composite packs. The literature underscores the importance of elemental ratios in shaping the microstructure, nodular formation, and overall properties of electrodeposited coatings [71,72]. Thus, tailoring these parameters is essential to optimize both the structural and functional properties of the coating for specific applications.

Figure S1 (Supplementary Materials) presents EDS mapping and the spectrum results for Ni-P and Ni-P-Cu coatings, providing insight into the elemental distributions of Nickel (Ni), Phosphorus (P), and Copper (Cu) within each sample. The elemental compositions correlate with SEM morphology images and reveal how variations in coating parameters affect the structure and element distribution in each sample. The Ni-P1 coating shows a smooth surface with small compact grains. This morphology is characteristic of the lower current density and shorter anode–cathode distance, which facilitates uniform deposition. The elemental mapping for Ni and P shows an even distribution, with no visible segregation or clustering, indicating that both elements are homogeneously distributed. Ni constitutes approximately 88.55 wt.%, while P is around 11.45 wt.% (Table S1). The higher Ni content aligns with the compact morphology, where reduced current density supports uniform deposition, allowing both Ni and P atoms to co-deposit evenly across the surface. The Ni-P2 sample has slightly larger grains than Ni-P1, resulting in a rougher surface morphology. This structure can be attributed to the increased current density and anode–cathode distance, promoting faster grain growth. The Ni and P mapping shows a uniform distribution similar to Ni-P1 but with slightly larger clusters, which correspond to the grainy morphology observed in SEM. Ni is around 86.3 wt.%, and P is 13.7 wt.% (Table S1), indicating a slight increase in P content compared to Ni-P1. This higher P content likely results from the faster deposition rate, which slightly favors P incorporation at the higher current density. The Ni-P3 coating exhibits the most pronounced granular structure among the Ni-P samples, with larger grains and increased surface roughness. Ni and P mapping show a consistent distribution but with more pronounced clustering, correlating with the larger grains observed in SEM images. Ni is approximately 85.9 wt.%, while P content increases to 14.1 wt.% (Table S1). The elevated P content at this higher current density (3 A/dm^2^) and longer distance may be attributed to the accelerated deposition rate, promoting P enrichment at the grain boundaries.

On the other hand, the Ni-P-Cu1 coating displays a mixed morphology with both small Ni-P grains and visible Cu particles. This structure is likely influenced by the co-deposition of Ni, P, and Cu at a relatively low current density and short anode–cathode distance. The mapping shows distinct distributions of Ni, P, and Cu, with Cu dispersed evenly across the surface, indicating successful incorporation. Ni constitutes 61.12 wt.%, P is 5.19 wt.%, and Cu is 33.69 wt.% (Table S1). The presence of Cu at this significant percentage supports a crystalline structure, as Cu deposition catalyzes more ordered grain formation. Ni-P-Cu2 shows a rougher morphology with larger grains, reflecting the increased current density and moderate anode–cathode distance, which enhances grain size. The distribution of Ni, P, and Cu remains uniform, with the Cu concentration slightly increased in certain regions, possibly due to localized accumulation. Ni is approximately 55.47 wt.%, P is 3.8 wt.%, and Cu is 40.73 wt.% (Table S1). The high Cu content further supports crystallinity, with the Cu catalyzing larger grain formations that match the SEM observations. Ni-P-Cu3 presents the roughest morphology, with irregularly distributed large grains and a relatively high concentration of Cu. This structure corresponds to the highest current density and longest anode–cathode distance. Ni, P, and Cu mappings show homogeneous distributions across the surface but with denser Cu regions, corresponding to areas of higher grain clustering. The Ni content drops to 47.38 wt.%, while P is 2.5 wt.% and Cu reaches 50.12 wt.% (Table S1). The high Cu concentration promotes crystalline growth and electrical conductivity, as Cu atoms integrate into the matrix, catalyzing the formation of larger more crystalline grains. The elemental analysis via EDS combined with SEM morphology highlights the influence of deposition parameters on the distribution and concentration of Ni, P, and Cu within the Ni-P and Ni-P-Cu coatings. In Ni-P coatings, the variation in current density and anode–cathode distance impacts the Ni to P ratio, with higher current densities favoring greater P incorporation, contributing to grain growth and surface roughness. In Ni-P-Cu coatings, the presence of Cu significantly alters the morphology, with Cu content increasing crystallinity and promoting larger grain structures. The highest Cu concentrations, as observed in Ni-P-Cu3, create a more crystalline and conductive structure, suitable for applications requiring high thermal and electrical conductivity. The choice of deposition parameters thus directly controls the coating morphology and elemental composition as stated in the literature [73,74], enabling tailored surface properties for specific application needs.

### 3.6. Phase Analysis of the PCM/EG Composite Packs with Ni-P and Ni-P-Cu Coatings

**Figure 7:**
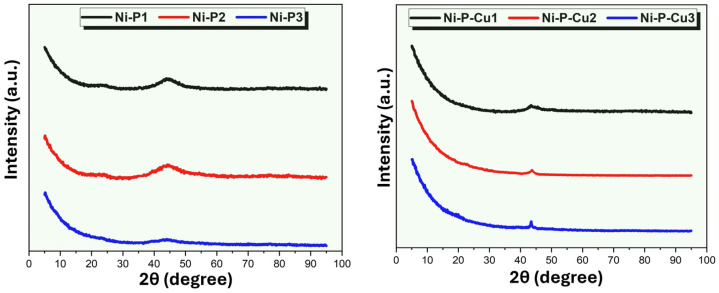
XRD patterns of the Ni-P- and Ni-P-Cu-coated PCM/EG composite packs.

Figure 7 displays the X-ray diffraction (XRD) patterns for the Ni-P and Ni-P-Cu coatings applied to PCM/EG composite packs, with the left panel showing patterns for Ni-P coatings (Ni-P1, Ni-P2, Ni-P3) and the right panel displaying patterns for Ni-P-Cu coatings (Ni-P-Cu1, Ni-P-Cu2, Ni-P-Cu3). These diffraction patterns provide insights into the phase composition and crystallinity of each coating type. The XRD pattern for Ni-P1 shows a broad hump, particularly around 2θ = 40–50°, characteristic of an amorphous or nanocrystalline Ni-P phase. The lack of sharp peaks indicates that the Ni-P1 coating does not possess a highly ordered crystalline structure, which is consistent with the conditions of low current density and short anode–cathode distance. These parameters favor a slower deposition rate, which can result in amorphous phases or very fine-grained nanocrystalline structures in the coating. The XRD pattern for Ni-P2 also displays a broad hump, similar to Ni-P1, but with slightly higher intensity. This suggests a slightly higher degree of ordering or partial crystallization within the Ni-P2 coating compared to Ni-P1. The increase in current density and anode–cathode distance likely accelerates the deposition rate, allowing for some degree of crystal formation but still maintaining an overall amorphous or nanocrystalline structure. The XRD pattern for Ni-P3 shows a broader hump with a slight shift toward lower 2θ angles. This feature indicates that Ni-P3 has the least crystalline structure among the Ni-P samples, likely due to the high current density and longer anode–cathode distance. These parameters promote a rapid deposition rate that hinders the formation of an ordered crystal structure, leading to a predominantly amorphous phase with only minimal crystallinity. Overall, the Ni-P coatings (Ni-P1, Ni-P2, and Ni-P3) exhibit predominantly amorphous or nanocrystalline structures. The broad peaks indicate limited crystalline domains within the coatings, with the degree of crystallinity decreasing as current density and anode–cathode distance increase.

The XRD pattern for Ni-P-Cu1 shows sharper peaks compared to the Ni-P coatings, with identifiable diffraction peaks around 2θ = 44°, corresponding to the (111) plane of crystalline copper (Cu). This sharp peak suggests that the incorporation of Cu in the Ni-P matrix increases the crystalline nature of the coating. The lower current density and shorter anode–cathode distance allow Cu to integrate into the matrix in a more organized manner, enhancing crystallinity. In the Ni-P-Cu2 sample, the XRD pattern displays an increase in peak intensity at 2θ = 44°, along with additional faint peaks. These indicate a more pronounced crystalline phase in the coating. The higher Cu content and moderate current density contribute to a more ordered structure, with Cu atoms promoting nucleation sites that facilitate the growth of crystalline domains. This behavior aligns with the morphology observed, where Cu facilitates grain growth and promotes a more crystalline coating structure. The XRD pattern for Ni-P-Cu3 shows the sharpest peaks among all samples, with intense diffraction at 2θ = 44° and additional peaks corresponding to crystalline Cu. The high current density and extended anode–cathode distance in this sample result in the rapid deposition of Cu, which further increases the crystallinity of the coating. This high crystallinity is consistent with the presence of large Cu grains observed in the SEM morphology. The elevated Cu content, along with rapid deposition, enhances the crystalline structure by promoting the formation of larger and more ordered grains within the Ni-P-Cu matrix. The XRD patterns provide clear evidence that the addition of Cu significantly enhances the crystallinity of the Ni-P coatings. In Ni-P coatings (Ni-P1, Ni-P2, and Ni-P3), the patterns are predominantly amorphous, indicating limited crystalline phases. The broad humps in these patterns suggest that the Ni-P coatings are either amorphous or contain very fine nanocrystals, which is expected under conditions that limit atomic mobility, such as high current densities and long anode–cathode distances. Conversely, the Ni-P-Cu coatings (Ni-P-Cu1, Ni-P-Cu2, and Ni-P-Cu3) exhibit sharper diffraction peaks, indicating the presence of a crystalline Cu phase within the Ni-P matrix. The addition of Cu introduces nucleation sites that promote crystallization, particularly evident in Ni-P-Cu3, where the highest current density and anode–cathode distance facilitate rapid Cu deposition, resulting in the most crystalline structure. This crystalline enhancement due to Cu incorporation is advantageous for applications requiring high thermal and electrical conductivity, as crystalline Cu provides improved conductivity pathways within the coating.

In conclusion, the XRD analysis confirms that the Ni-P coatings primarily exhibit amorphous structures, while the Ni-P-Cu coatings exhibit a mixed structure with a significant crystalline Cu phase. The degree of crystallinity within Ni-P-Cu coatings is directly correlated with the Cu content, which increases with current density and anode–cathode distance, supporting the formation of larger and more ordered crystalline domains. These findings align with the SEM observations and EDS results, highlighting the role of Cu in enhancing structural order within the composite coatings. The addition of Cu to Ni-P coatings and its impact on the crystalline structure and properties is a frequently discussed topic in the literature [47,75,76].

**Figure 8:**
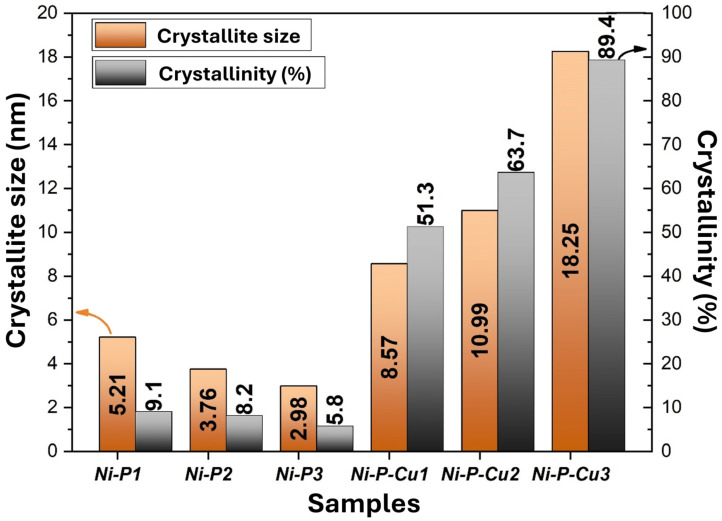
Crystallite size and crystallinity values of the PCM/EG composite packs with Ni-P and Ni-P-Cu coatings obtained under different parameters.

Figure 8 presents the crystallite size (in nanometers) and crystallinity percentage of each coating sample, showing the structural differences between Ni-P and Ni-P-Cu coatings. The Ni-P samples (Ni-P1, Ni-P2, Ni-P3) exhibit relatively small crystallite sizes, ranging from 2.98 to 5.21 nm, and low crystallinity percentages, with values between 5.8% and 9.1%. These results align with the predominantly amorphous structures observed in the XRD patterns, where broad humps indicated limited crystalline domains. The trend shows that as the current density and anode–cathode distance increase (from Ni-P1 to Ni-P3), crystallite size decreases and crystallinity percentage remains low, suggesting that higher deposition rates limit the formation of ordered structures in Ni-P coatings. In contrast, the Ni-P-Cu samples (Ni-P-Cu1, Ni-P-Cu2, Ni-P-Cu3) demonstrate significantly larger crystallite sizes and higher crystallinity percentages. The crystallite sizes range from 8.57 nm for Ni-P-Cu1 to 18.25 nm for Ni-P-Cu3, while the crystallinity percentages increase from 51.3% to 89.4%. This increase in structural order can be attributed to the incorporation of Cu, which acts as a nucleating agent, promoting the growth of crystalline domains within the Ni-P matrix. The highest crystallinity and crystallite size observed in Ni-P-Cu3 reflects the combined effect of high current density and greater anode–cathode distance, which enhance Cu deposition and lead to more ordered and larger crystalline domains. These trends indicate that the addition of Cu and the adjustment of deposition parameters significantly enhance the crystalline structure of the coatings, making Ni-P-Cu coatings, especially Ni-P-Cu3, more suitable for applications requiring high thermal and electrical conductivity due to their improved crystallinity.

Based on the data analysis, Ni-P1 and Ni-P-Cu3 are expected to exhibit the most favorable coating characteristics among the samples. For the Ni-P series, Ni-P1 shows a compact and uniform morphology with relatively small crystallite size (5.21 nm) and moderate crystallinity (9.1%). This structure is anticipated to enhance its performance in thermal management applications, as the fine compact grain structure should contribute to stability under thermal cycling. The smaller grain size in Ni-P1 is likely to reduce the potential for thermal stress accumulation, helping to maintain structural integrity and adhesion under varying thermal conditions. Furthermore, the relatively amorphous nature with moderate crystallinity in Ni-P1 is expected to provide better thermal stability, which is essential for reliable performance in thermal applications. For the Ni-P-Cu series, Ni-P-Cu3 is anticipated to exhibit superior properties, given its large crystallite size (18.25 nm) and high crystallinity (89.4%), which suggest a highly ordered crystalline structure. The presence of Cu likely promotes nucleation, resulting in increased crystallinity and larger more defined crystalline domains. Such a structure is expected to be advantageous for applications requiring high electrical and thermal conductivity, and a well-ordered crystal lattice generally facilitates efficient pathways for electron and heat transfer. The morphology of Ni-P-Cu3, with its larger grains and high crystallinity, should further enhance its suitability for thermal and electrical applications, offering improved stability and conductivity compared to more amorphous coatings. Mechanically, Ni-P1’s compact and fine-grained structure is likely to provide superior wear resistance, as smaller grain boundaries typically reduce the risk of crack initiation under mechanical stress. For Ni-P-Cu3, the increased crystallinity and grain size are expected to improve hardness and toughness, qualities that are beneficial for coatings subjected to mechanical load. The higher Cu content in Ni-P-Cu3 is also anticipated to contribute to mechanical strength, providing a robust crystalline matrix that can better resist deformation under stress. Consequently, Ni-P1 and Ni-P-Cu3 are identified as the most promising candidates for their respective categories, and future studies will likely focus on these coatings for comparative performance assessments. These coatings are expected to serve as benchmarks in further research, where their thermal, electrical, and mechanical properties can be optimized for specific applications. In the literature, it is frequently noted that crystalline coatings generally provide superior thermal conductivity and enhanced mechanical properties compared to amorphous coatings. Studies consistently demonstrate that crystalline structures facilitate better heat transfer and exhibit improved durability, hardness, and wear resistance, making them favorable for applications requiring high-performance thermal and mechanical characteristics [77,78,79].

### 3.7. Mechanical Properties of the PCM/EG Composite Packs with Ni-P and Ni-P-Cu Coatings

**Figure 9:**
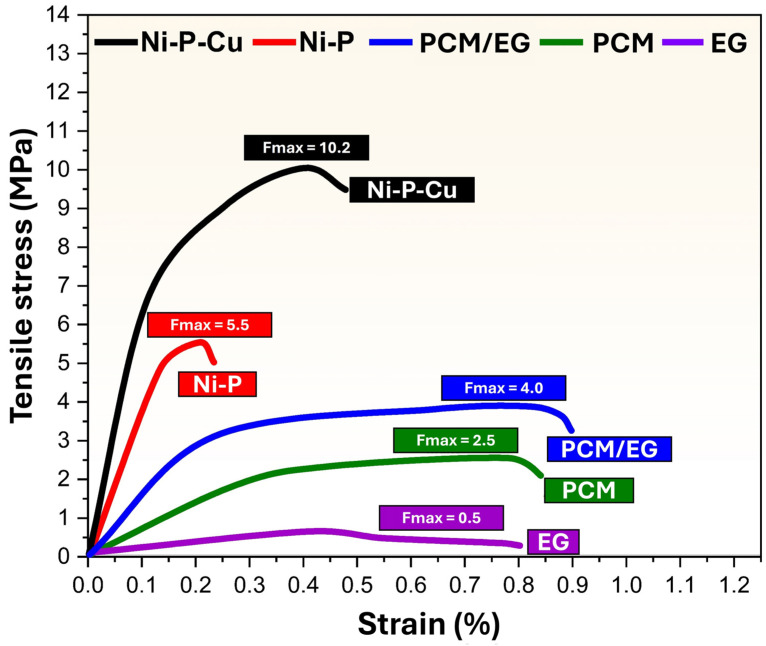
Tensile stress–strain curves of the samples.

Figure 9 shows the tensile stress–strain behavior of different materials, including Ni-P-coated PCM/EG composite packs, Ni-P-Cu-coated PCM/EG composite packs, PCM/EG composite packs, pure PCM, and pressed EG blocks. The tensile stress is plotted against strain, with maximum tensile strength values (F_max_) indicated for each sample. The Ni-P-Cu-coated PCM/EG sample exhibits the highest tensile strength among all samples, with a maximum stress of F_max_ = 10.2 MPa at a strain of approximately 0.22%. This high tensile strength suggests that the Ni-P-Cu coating substantially enhances the mechanical performance of the PCM/EG composite. The addition of Cu to the Ni-P matrix increases crystallinity and provides a rigid and cohesive structure, which is reflected in the material’s ability to withstand high stress with minimal strain. The Ni-P-coated PCM/EG composite achieves a maximum tensile strength of F_max_ = 5.5 MPa at around 0.18% strain. Although this strength is lower than that of the Ni-P-Cu-coated sample, it is still significantly higher than the uncoated PCM/EG composite. The Ni-P layer improves the mechanical stability of the PCM/EG composite by creating a more compact and cohesive structure, although it lacks the additional reinforcement provided by Cu, which contributes to the comparatively lower strength. The uncoated PCM/EG composite has a maximum tensile strength of F_max_ = 4.0 MPa at a strain of about 0.7%. This composite demonstrates moderate mechanical strength with a higher strain tolerance than the coated samples, indicating some flexibility. The PCM matrix contributes to this flexibility, while the EG particles provide structural reinforcement. However, the absence of a coating results in lower overall tensile strength compared to the coated versions. The pure PCM sample reaches a maximum tensile strength of F_max_ = 2.5 MPa at approximately 0.6% strain. Without reinforcement from EG particles or any surface coating, PCM alone shows limited mechanical strength and is relatively flexible. This low tensile strength indicates that PCM by itself may not be suitable for applications requiring substantial load-bearing capacity, although its flexibility could be advantageous in certain thermal applications. The pressed EG sample exhibits the lowest tensile strength, with F_max_ = 0.5 MPa at around 0.2% strain. This low strength is expected due to the lack of bonding within the EG particles, which results in a fragile structure. While EG is beneficial as a thermally conductive filler, its standalone mechanical properties are insufficient for load-bearing applications.

The results indicate that Ni-P-Cu-coated PCM/EG has the highest mechanical strength among the samples, attributed to the dual enhancement from Ni-P and Cu. The Cu component improves crystallinity, which strengthens the overall structure, providing rigidity and stability under mechanical stress. This makes Ni-P-Cu-coated PCM/EG ideal for applications that require both thermal management and high mechanical durability. The Ni-P-coated PCM/EG also demonstrates a considerable increase in tensile strength compared to the uncoated composite, as the Ni-P coating enhances the structural integrity of the PCM/EG matrix. However, without the additional crystalline reinforcement from Cu, the Ni-P coating offers moderate strength, making it suitable for applications where moderate load-bearing capacity is sufficient. The uncoated PCM/EG composite, while demonstrating a balance between flexibility and strength, lacks the structural enhancements of the coated samples. It may be suitable for applications that prioritize thermal properties over mechanical strength, given its moderate tensile strength and higher strain tolerance. Pure PCM and pressed EG have the lowest tensile strengths, highlighting their limitations as standalone materials for structural applications. PCM’s flexibility could be advantageous in applications where some deformability is acceptable, while EG, although beneficial for its thermal conductivity, lacks the necessary mechanical cohesion for load-bearing use. Finally, Ni-P-Cu-coated PCM/EG and Ni-P-coated PCM/EG show the most promising mechanical properties for applications requiring tensile strength and rigidity. Future studies may focus on these coatings to further explore their performance in thermomechanical applications, with Ni-P-Cu-coated PCM/EG serving as a benchmark for high-strength and thermally conductive coatings.

**Figure 10:**
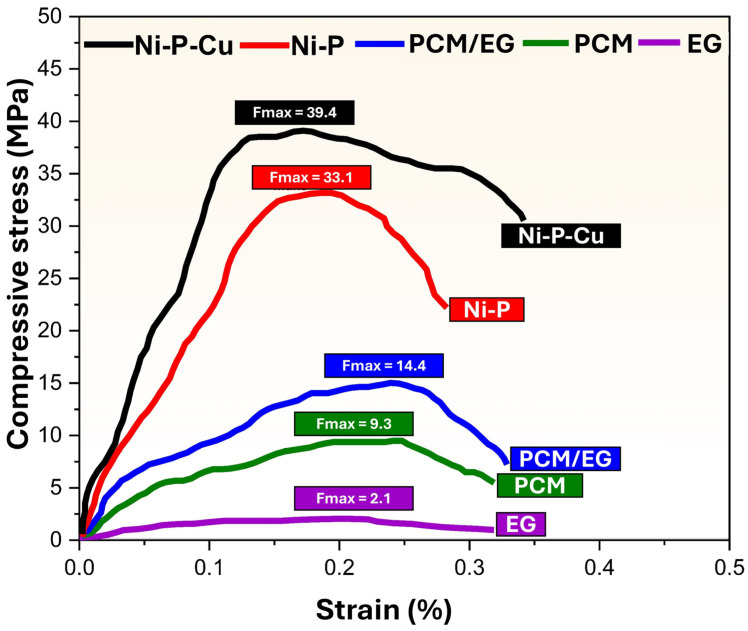
Compressive stress–strain curves of the samples.

Figure 10 illustrates the compressive stress–strain behavior of various materials, including Ni-P-coated PCM/EG, Ni-P-Cu-coated PCM/EG, PCM/EG composite, pure PCM, and pressed EG. The Ni-P-Cu-coated PCM/EG exhibits the highest maximum compressive strength, reaching Fmax = 39.4 MPa at a strain of approximately 0.25%. This high compressive strength is attributed to the presence of Cu, which reinforces the structure by increasing the crystallinity and mechanical integrity of the Ni-P coating. The Ni-P-Cu coating creates a rigid and stable network that effectively distributes the compressive load, preventing localized deformation and enhancing the load-bearing capacity under compression. The Ni-P-coated PCM/EG composite has a compressive strength of Fmax = 33.1 MPa at around 0.23% strain. This strength, although slightly lower than Ni-P-Cu, still demonstrates a significant improvement over the uncoated PCM/EG composite. The Ni-P layer adds structural integrity by forming a dense compact coating around the PCM/EG structure, providing resistance to compressive forces. However, the lack of Cu limits the crystallinity, which results in slightly lower compressive strength compared to Ni-P-Cu. The uncoated PCM/EG composite shows a maximum compressive strength of Fmax = 14.4 MPa at a strain of about 0.27%. This intermediate compressive strength reflects the structural support provided by the EG particles within the PCM matrix, allowing it to absorb and distribute compressive loads to a certain extent. However, without the protective coating, the PCM/EG composite is more susceptible to localized deformation and has reduced strength compared to the coated versions. Pure PCM achieves a compressive strength of Fmax = 9.3 MPa at approximately 0.24% strain. PCM alone provides a limited compressive resistance, as it lacks both reinforcement from EG particles and protective coatings. The relatively low compressive strength and moderate strain tolerance indicate that PCM may deform more easily under load, making it less suitable for applications requiring high compressive strength. The EG sample has the lowest maximum compressive strength, reaching Fmax = 2.1 MPa at around 0.12% strain. This minimal strength can be attributed to the weak interparticle bonding within the EG structure, which leads to a highly porous and fragile material under compression. While EG is valuable for its thermal conductivity properties, its standalone structure is insufficiently robust to withstand compressive forces effectively. The enhanced compressive strength of the Ni-P-Cu-coated PCM/EG sample is a result of the Ni-P-Cu layer’s crystalline structure, which adds rigidity and cohesion to the composite. The Cu addition within the Ni-P matrix promotes crystallization, which strengthens the interatomic bonding and helps to evenly distribute the compressive force throughout the material. This makes Ni-P-Cu-coated PCM/EG an excellent candidate for applications where both high compressive strength and structural stability are required. The Ni-P-coated PCM/EG, while lacking the additional reinforcement provided by Cu, still shows a substantial increase in compressive strength. The dense Ni-P layer around the PCM/EG structure adds resistance to compression by forming a compact cohesive coating. This improved structural integrity makes Ni-P-coated PCM/EG suitable for applications that demand moderate compressive strength but do not require the maximum performance of Ni-P-Cu. For the uncoated PCM/EG composite, the presence of EG particles provides a basic level of reinforcement, allowing the PCM/EG composite to resist compressive forces to some degree. However, without the enhanced cohesion from the coating, the PCM/EG structure is less effective at handling high compressive loads, as the EG particles may separate or shift under stress, leading to lower compressive strength. Pure PCM and pressed EG samples show significantly lower compressive strengths. PCM, though able to absorb strain moderately well, lacks reinforcement, making it prone to deformation under compression. The pressed EG sample’s low compressive strength highlights its fragility in bulk form, as the loose particle structure is unable to resist compressive forces effectively. In conclusion, Ni-P-Cu-coated PCM/EG and Ni-P-coated PCM/EG demonstrate the highest compressive strengths, with Ni-P-Cu outperforming all other samples due to the structural reinforcement provided by Cu. These materials are expected to perform well in applications requiring high compressive strength and mechanical stability. Many studies underscore the effectiveness of copper in reinforcing the mechanical attributes of Ni-P coatings, making them highly suitable for applications where enhanced durability and mechanical stability are required [42,80,81]. Moreover, in many studies in the literature, the improvement in mechanical properties in similar structures has been achieved through the use of polymer matrices. However, the use of polymer matrices significantly reduces thermal conductivity. For example, Yang et al. [82] reported that the strength of an EG/PCM structure was approximately 7 MPa with a thermal conductivity of about 8 W/m·K. While the use of a polymer matrix increased strength to over 8 MPa, it caused a reduction of approximately 30% in thermal conductivity. In contrast, the current study demonstrates that high thermal conductivity coatings not only enhanced the mechanical strength but also improved thermal conductivity performance.

### 3.8. Thermal Conductivity of the PCM/EG Composite Packs with Ni-P and Ni-P-Cu Coatings

**Figure 11:**
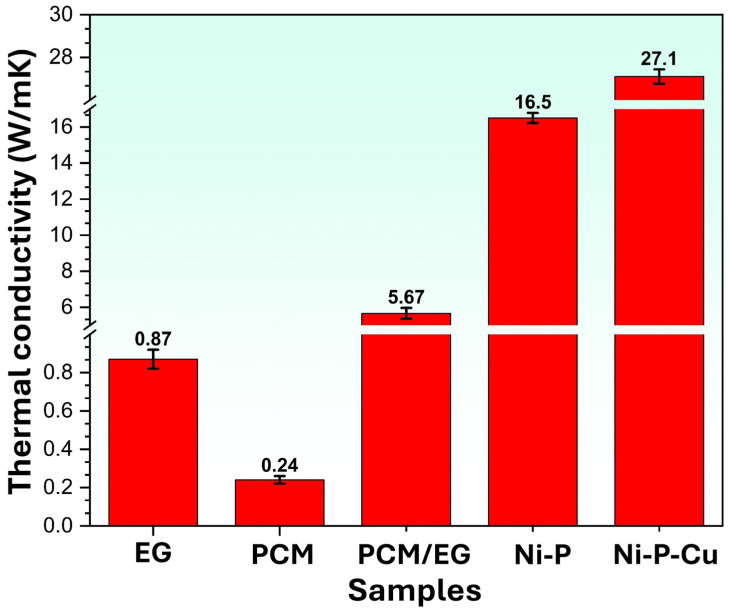
Thermal conductivity of the samples.

In Figure 11, the thermal conductivity values for pure PCM, expanded graphite (EG), PCM/EG composite, Ni-P-coated PCM/EG, and Ni-P-Cu-coated PCM/EG are displayed, showcasing the enhancement in thermal conductivity through different material modifications and coatings. Pure PCM exhibits the lowest thermal conductivity at approximately 0.24 W/m·K, which can be attributed to its molecular structure and low density. The crystalline structure and weak atomic bonds in paraffin-based PCM limit its ability to conduct heat effectively, making it a poor thermal conductor. On the other hand, expanded graphite shows a thermal conductivity of around 0.87 W/m·K. This relatively higher conductivity is due to the strong covalent bonds between carbon atoms and the planar arrangement of graphite sheets, which facilitate phonon movement and enable efficient in-plane heat transfer. However, the porous structure of expanded graphite, with nearly 90% porosity, restricts its overall thermal conductivity by limiting continuous pathways for heat transfer. When PCM is impregnated into expanded graphite to form a PCM/EG composite, the thermal conductivity rises to about 5.67 W/m·K. This improvement is largely due to the PCM filling the porous structure of graphite, thereby creating additional points of contact between graphite particles and enhancing heat conduction pathways within the composite. The PCM material not only fills the voids but also brings its phase-change capability, allowing it to absorb and store latent heat, which contributes to better thermal stability and more effective heat dissipation. Applying a Ni-P coating onto the PCM/EG composite further increases its thermal conductivity to approximately 16.5 W/m·K. The Ni-P coating, with its metallic nature, introduces effective heat transfer pathways due to the strong metallic bonds that facilitate rapid heat conduction. The presence of phosphorus in the coating provides additional structural stability, ensuring a uniform and continuous heat-conductive layer around the PCM/EG composite. This metallic coating significantly improves the ability of the composite to dissipate heat under thermal load. The highest thermal conductivity, measured at 27.1 W/m·K, is achieved by the Ni-P-Cu-coated PCM/EG composite. The addition of copper, a metal with high intrinsic thermal conductivity, further enhances heat transfer within the coating by optimizing the crystalline structure. Copper atoms improve the efficiency of phonon transport and minimize dislocation movement, which allows for more efficient heat conduction across the composite. This high thermal conductivity in the Ni-P-Cu-coated PCM/EG composite makes it particularly suitable for applications in thermal management, such as battery systems, where efficient heat dissipation is essential for preventing overheating and ensuring operational safety. The enhancement in thermal conductivity through these modifications is supported by multiple mechanisms. The Ni-P and Ni-P-Cu coatings provide continuous metallic pathways that facilitate thermal energy transfer far more efficiently than uncoated composites. Expanded graphite’s natural high thermal conductivity due to its hexagonal carbon arrangement is leveraged within the PCM/EG composite, where PCM fills the graphite pores and creates closer contact between particles, enhancing phonon transport. Additionally, PCM’s phase-change properties contribute to thermal stability by absorbing and releasing heat as it transitions between phases. While studies in the literature [57,65,83,84] show that the thermal conductivity of EG/PCM composites with similar ratios of EG and PCM typically ranges between approximately 1 and 10 W/m·K, this study clearly demonstrates the significant improvement achieved through Ni-P and Ni-P-Cu surface coatings, with values increasing up to approximately 27 W/m·K. The combination of these structural and material modifications, along with the protective and conductive metallic coatings, results in a highly effective heat management solution, ideal for applications that demand both thermal and structural performance.

### 3.9. Thermal Management Properties of the PCM/EG Composite Packs with Ni-P and Ni-P-Cu Coatings

**Figure 12:**
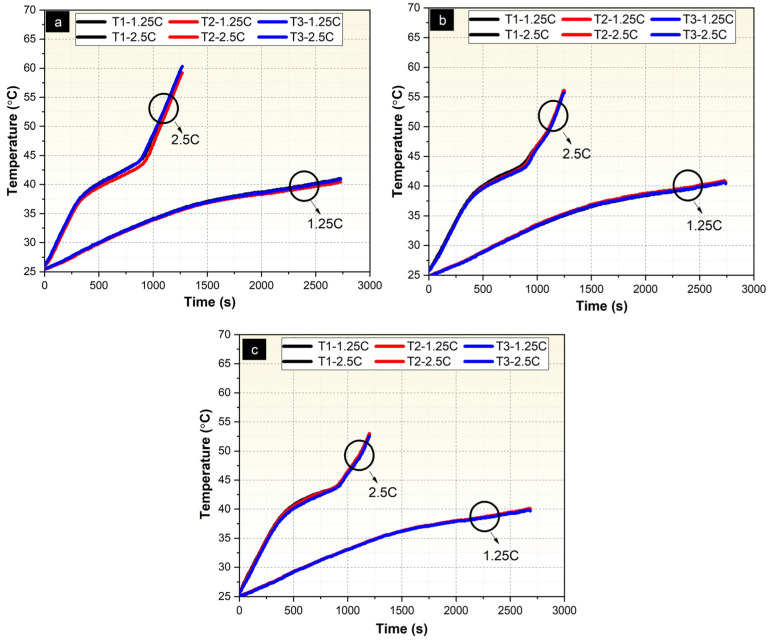
Local temperature variations for (**a**) PCM/EG, (**b**) Ni-P, and (**c**) Ni-P-Cu.

**Figure 13:**
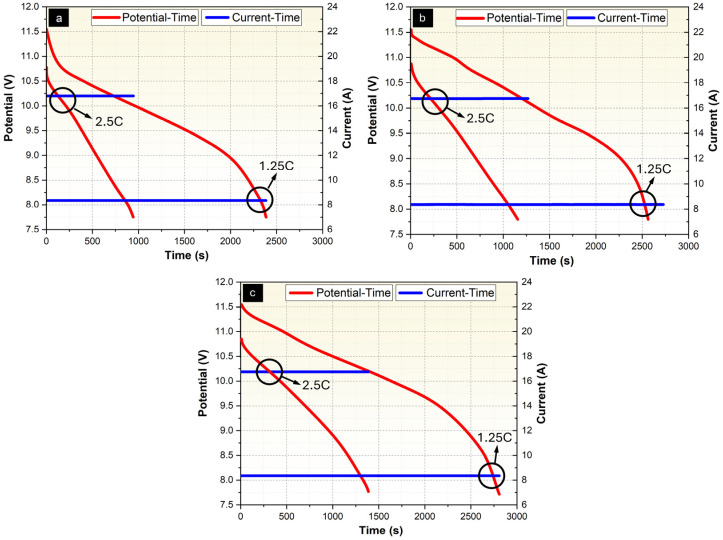
Time-dependent variation in the potential value for (**a**) PCM/EG, (**b**) Ni-P, and (**c**) Ni-P-Cu.

**Figure 14:**
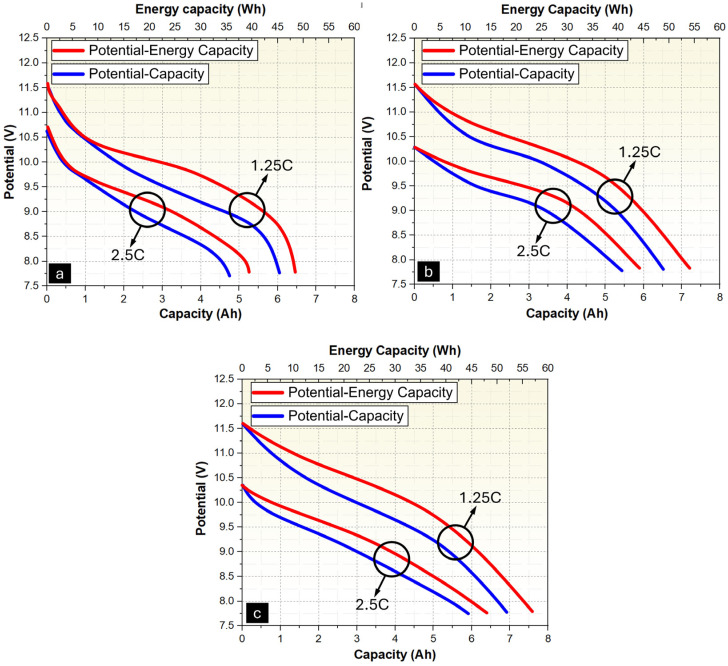
Discharge capacity and energy capacity variation for (**a**) PCM/EG, (**b**) Ni-P, and (**c**) Ni-P-Cu.

In addition to enhancing thermal properties, these types of coatings significantly improve the mechanical characteristics of PCM/EG composites. The application of Ni-P and Ni-P-Cu coatings not only increases the composite’s resistance to thermal degradation but also strengthens its structural integrity under mechanical loads. By promoting crystallinity and forming a cohesive rigid layer around the PCM/EG matrix, these coatings enhance both compressive and tensile strength, making the composite more resilient and suitable for demanding applications where both thermal management and mechanical durability are critical. Studies show that Ni-P and Ni-P-Cu coatings can improve the thermal conductivity of substrates, a beneficial property for applications requiring efficient heat dissipation. The addition of Cu in particular enhances the thermal performance of the Ni-P matrix, as copper has high intrinsic thermal conductivity and, when alloyed with Ni-P, forms a crystalline structure that further facilitates heat transfer [80,85,86]. In this study, the experimental setup was designed to monitor temperature variations at specific points, with the initial temperatures at time t = 0 set to 25 °C to achieve thermal equilibrium. Once stability was ensured, the battery packs were loaded via an electronic load unit at discharge rates of 1.25C and 2.5C. Two operational limits were defined based on battery characteristics: a cut-off voltage of 7.8 V and a maximum temperature of 60 °C. Experiments were terminated as soon as either of these values was reached. Performance tests for different PCM/EG matrix configurations (PCM/EG packs, Ni-P-coated PCM/EG packs, and Ni-P-Cu-coated PCM/EG packs) were conducted under both discharge rates. The results are presented in terms of local temperature profiles (Figure 12), voltage decay over time (Figure 13), and capacity (Ah) and energy capacity (Wh) variations (Figure 14).

In Figure 12, the local temperature profiles for different configurations indicate a characteristic pattern during the discharge process. Initially, there is a linear temperature increase, followed by a period where the rate of temperature rise decreases, and finally, another phase with a linear increase in temperature. The second phase, where the temperature rises slowly, is related to the phase change temperature of the composite material (RT-42, approximately 38–43 °C), during which the PCM absorbs latent heat in the solid-to-liquid transition. This phase change allows the composite structure to absorb the heat generated within the battery pack, thereby significantly suppressing the temperature increase. Once the phase transition is complete, the rate of temperature rises resumes due to sensible heat storage. At lower discharge rates, the third phase is not observed because the phase transition is incomplete. Figure 12a illustrates the temperature variation in PCM/EG battery packs under 1.25C and 2.5C discharge rates. The initial phase is characterized by a steady increase in temperature, with a noticeable plateau around 38 °C to 43 °C. This corresponds to the phase change process of the PCM, where the material absorbs latent heat during the solid-to-liquid transition. At 2.5C, the phase changes complete faster, resulting in a steeper temperature increase afterward due to the higher discharge rate and faster heat generation from internal resistance. Figure 12b depicts the temperature evolution in Ni-P-coated PCM/EG battery packs for the same discharge rates. Compared to PCM/EG packs, the Ni-P coating enhances thermal conductivity, leading to a slightly more efficient heat distribution during the discharge process. The latent heat absorption phase (38–43 °C) is more pronounced and lasts longer under both discharge rates, especially at 1.25C, indicating improved thermal management due to the Ni-P coating. Figure 12c presents the temperature profiles for Ni-P-Cu-coated PCM/EG packs under 1.25C and 2.5C discharge rates. The addition of Cu to the coating further improves thermal conductivity and heat dissipation. This results in the slowest temperature rise among all configurations. At 2.5C, the PCM undergoes phase change more efficiently, and the temperature plateau during the latent heat absorption phase is extended. The overall peak temperature is also lower compared to PCM/EG and Ni-P-coated PCM/EG packs, highlighting the superior thermal performance of this configuration. Considering the highest temperature levels reached in the same period, it can be noted that the temperature increases steadily, reaching a peak of approximately 43 °C before stabilizing at 1.25C for PCM/EG packs (Figure 12a). The plateau corresponds to the phase change temperature range of the PCM (38 –43 °C), where the PCM absorbs latent heat during the solid-to-liquid transition. At 2.5C, the temperature rises faster, peaking at approximately 61 °C. The higher discharge current accelerates the heat generation, and the phase change completes earlier, leading to a steeper rise in temperature after the phase transition for PCM/EG packs as seen in Figure 12a. Figure 12b displays the temperature behavior of Ni-P-coated PCM/EG battery packs under the same discharge rates. At 1.25C, the temperature reaches approximately 41 °C at the phase change plateau and peaks at around 45 °C. The Ni-P coating enhances thermal conductivity, leading to better heat management and a slightly lower peak temperature than uncoated PCM/EG packs. At 2.5C, the temperature increases more rapidly but peaks at a slightly lower value of approximately 57 °C, indicating improved thermal performance due to the Ni-P coating. The phase change plateau remains evident, suggesting effective latent heat absorption. Figure 12c illustrates the temperature variation in Ni-P-Cu-coated PCM/EG battery packs under 1.25C and 2.5C discharge rates. At 1.25C, the temperature stabilizes at the phase change plateau around 39 °C and peaks at 42 °C, the lowest peak among all configurations. The addition of Cu further improves heat dissipation and thermal conductivity, resulting in better temperature control. At 2.5C, the temperature rises faster but stabilizes at a peak of approximately 53 °C, which is significantly lower than the peaks observed in the PCM/EG and Ni-P-coated PCM/EG packs. This configuration demonstrates superior thermal performance, effectively managing the heat generated even at high discharge rates. Similarly, the addition of copper to enhance thermal conductivity has been applied in studies in the literature, effectively delaying the time required to reach the critical temperature [87,88].

In Figure 13, the time-dependent voltage decay is displayed for PCM/EG, Ni-P-coated PCM/EG, and Ni-P-Cu-coated PCM/EG packs under both discharge rates. The discharge profiles of PCM/EG battery packs under 1.25C and 2.5C rates are presented in Figure 13a. For the 1.25C discharge rate, the voltage gradually decreases from 11.5 V to around 7.5 V, with the discharge lasting approximately 2400 s. During this period, the current trend remains constant at 8.3 A, reflecting stable performance over the discharge cycle. This relatively longer duration highlights the moderate energy output capacity of the PCM/EG pack at lower current rates. In contrast, for the 2.5C rate, the voltage drops significantly faster, resulting in a shorter discharge duration of about 900 s, while the current stabilizes at 10.1 A. The rapid voltage decline reflects the limited thermal and electrochemical management capabilities of the PCM/EG pack under higher currents. Figure 13b shows the voltage and current profiles of Ni-P-coated PCM/EG battery packs. At 1.25C, the coating improves the discharge duration to approximately 2600 s, compared to the uncoated PCM/EG pack. The voltage shows a controlled decline from 11.5 V to 7.5 V, while the current remains steady at 8.3 A. The Ni-P coating enhances electrochemical stability, leading to better utilization of the energy stored in the battery. When subjected to 2.5C, the discharge duration increases to 1200 s—a noticeable improvement over the PCM/EG pack. Although the current is maintained at 10.1 A, the voltage curve indicates that the Ni-P coating helps in reducing the rate of voltage drop, likely due to better surface conductivity and reduced internal resistance. The performance of Ni-P-Cu-coated PCM/EG packs is depicted in Figure 13c. Under the 1.25C rate, the discharge lasts for about 2800 s, which is longer than both the PCM/EG and Ni-P-coated packs. The voltage transitions smoothly from 11.5 V to 7.5 V, while the current remains stable at 8.3 A. The additional Cu layer not only enhances thermal conductivity but also improves charge transfer efficiency, resulting in prolonged discharge duration. At the 2.5C rate, the pack demonstrates the best performance among the three configurations, with a discharge duration of 1400 s. The voltage decline is slower compared to the other cases, further highlighting the advantages of the Ni-P-Cu coating in managing high discharge currents by improving thermal and electrochemical stability. Consequently, The Ni-P-Cu-coated PCM/EG pack achieves the best results, delivering longer discharge times and more stable voltage profiles. This configuration effectively handles both thermal management and electrochemical stability, making it superior for high-performance applications.

Figure 14 presents the relationship between voltage decay, discharge capacity (Ah), and energy capacity (Wh) for each configuration under the two discharge rates. The voltage-capacity and voltage-energy capacity relationships for the PCM/EG pack are shown in Figure 14a. The energy capacity reaches approximately 50 Wh, while the total capacity is near 6 Ah, showing moderate energy retention at 1.25C. The voltage declines steadily from 11.5 V to 7.5 V, illustrating the pack’s limited ability to sustain high energy output at the lower discharge rate. At 2.5C, the energy capacity decreases significantly to around 30 Wh, while the capacity reaches just above 4 Ah. The steep decline in voltage, coupled with the reduced energy output, highlights the thermal and electrochemical limitations of PCM/EG packs under high discharge currents. Figure 14b presents the performance of Ni-P-coated PCM/EG packs. For the 1.25C discharge rate, the energy capacity improves to 55 Wh, and the capacity increases to 6.5 Ah. The voltage profile shows a smoother decline compared to the uncoated PCM/EG pack, reflecting enhanced energy retention due to the Ni-P coating. At 2.5C, the energy capacity increases to approximately 40 Wh, with the total capacity reaching nearly 5 Ah. The slower voltage decline indicates that the Ni-P coating mitigates thermal and electrical losses, enabling improved performance at higher discharge rates. The Ni-P-Cu-coated PCM/EG pack results are depicted in Figure 14c. The energy capacity further improves to nearly 58 Wh, with the total capacity reaching 6.8 Ah for the rate of 1.25C. The voltage profile remains stable for a longer duration, emphasizing the enhanced charge transfer and thermal conductivity provided by the Cu coating. The energy capacity is maintained at around 45 Wh, while the capacity reaches 5.5 Ah at 2.5C. This configuration demonstrates the slowest voltage decline among the three, highlighting the superior performance of the Ni-P-Cu coating in sustaining high energy output under demanding conditions.

Across all configurations, the Ni-P-Cu coating achieves the best energy and capacity results, followed by the Ni-P coating and PCM/EG pack. This trend is consistent with the improved thermal and electrochemical stability provided by the successive coatings. The Ni-P coating enhances surface conductivity, reduces internal resistance, and provides a more uniform current distribution, which prevents localized overheating and voltage drops. The addition of Cu further improves thermal management by dissipating heat more effectively, reducing the impact of Joule heating during high discharge rates. These improvements are evident in both energy capacity (Wh) and total capacity (Ah), particularly under higher discharge rates. The findings align with earlier observations of prolonged discharge times and controlled temperature profiles, solidifying the effectiveness of the Ni-P and Ni-P-Cu coatings in optimizing battery performance.

## 4. Conclusions

This study investigated the thermal, mechanical, and electrochemical performance of PCM/EG composites with Ni-P and Ni-P-Cu coatings under various operational conditions to enhance battery thermal management systems. Based on the experimental findings and detailed analyses, the following conclusions were drawn:

1. The Ni-P and Ni-P-Cu coatings exhibited a highly crystalline microstructure, with Ni-P-Cu coatings showing the highest degree of crystallinity (89%) among the tested samples. This enhanced crystallinity improves thermal and mechanical properties by providing more uniform and stable pathways for heat and stress distribution. The crystalline structure on Ni-P-Cu coating reduces thermal resistance and improves the mechanical stability of the PCM/EG composite, allowing it to effectively manage heat and withstand mechanical stresses during battery operation. This structural integrity is crucial for applications requiring both high thermal conductivity and durability under cyclic loading conditions;
2. The Ni-P-Cu-coated PCM/EG composites exhibited the highest thermal conductivity at 27.1 W/m·K, significantly outperforming both the uncoated PCM/EG (5.67 W/m·K) and Ni-P-coated PCM/EG (16.5 W/m·K) configurations. This enhancement is attributed to the superior heat conduction pathways provided by the Ni-P-Cu metallic coating, which benefits from both the high thermal conductivity of Cu and the stable structure of the Ni-P matrix. This makes Ni-P-Cu-coated PCM/EG composites highly suitable for applications demanding efficient thermal management;
3. The phase-change temperatures of the PCM/EG composites between 38 °C and 43 °C allowed effective latent heat absorption, which suppressed temperature rise during the solid-to-liquid transition phase. This effect was most pronounced under a 1.25C discharge rate, where the composites demonstrated prolonged thermal stability, effectively delaying temperature increase during battery operation;
4. The Ni-P-Cu-coated PCM/EG composite also demonstrated the highest compressive strength, reaching up to 39.4 MPa, and tensile strength, indicating its potential to withstand mechanical stress in battery packs. These improved mechanical properties can be attributed to the cohesive and crystalline structure provided by the Cu-rich Ni-P-Cu coating, enhancing structural integrity alongside thermal performance;
5. At 2.5C, the Ni-P-Cu-coated PCM/EG stabilized at approximately 53 °C after ~1300 s, while the PCM/EG reached ~61 °C within the same duration, demonstrating improved thermal regulation for Sample C. At 1.25C, the PCM/EG exhibited a temperature of approximately 42 °C after ~2700 s, whereas the Ni-P-Cu-coated PCM/EG stabilized at ~40 °C, highlighting enhanced thermal performance with the coating;
6. In the 2.5C discharge test, the Ni-P-Cu-coated PCM/EG maintained the voltage above 8.0 V for approximately 1400 s, while the PCM/EG dropped to 8.0 V within 900 s, indicating a 55% improvement in discharge duration. Similarly, at 1.25C, the Ni-P-Cu-coated PCM/EG reached 8.0 V in about 2800 s, compared to 2400 s for PCM/EG, showing a 17% improvement. This demonstrates that the Ni-P-Cu coating significantly reduces the rate of voltage drop, enhancing the stability of the battery under both test conditions;
7. The Ni-P and Ni-P-Cu coatings substantially improved the performance of PCM/EG composites in terms of capacity and energy retention, particularly under higher C-rate conditions. Ni-P-coated packs showed a 14% increase in energy capacity at 2.5C compared to PCM/EG, reaching approximately 35 Wh, while at 1.25C, it achieves a 10% improvement with 55 Wh. Ni-P-Cu-coated packs demonstrated the best performance, with a 33% increase in energy capacity at 2.5C, reaching approximately 40 Wh, and a 20% improvement at 1.25C, reaching 60 Wh. This progressive enhancement highlights the effectiveness of these coatings in enhancing energy storage capabilities;
8. The Ni-P-Cu-coated PCM/EG samples retained higher discharge capacities and energy capacities at both discharge rates compared to uncoated samples, maintaining operational efficiency and prolonging cycle life. However, increased discharge rates generally led to greater capacity losses due to the limitations of ion mobility and higher internal resistance for all packs;
9. This work demonstrates that Ni-P and Ni-P-Cu coatings on PCM/EG composites offer substantial improvements in thermal, mechanical, and electrochemical properties, making them promising candidates for efficient and durable thermal management in energy storage applications;
10. Despite the improvements, the coatings add additional weight and cost, which could impact applications sensitive to weight or budget constraints. Additionally, higher thermal and mechanical stability might still not fully counteract the effects of rapid prolonged high discharge rates (e.g., above 2.5C), as observed with slight capacity losses due to limitations in ion transport. Future work should explore multi-layer coatings and the integration of additional conductive fillers to further enhance both thermal and mechanical properties without significant weight penalties. Studies on the long-term durability of these coatings under repeated charge-discharge cycles would provide valuable insights into the longevity and practical application of such materials in advanced battery systems. The mechanical and thermal properties of the coated EG/PCM composites were not evaluated under harsh environmental conditions, which is crucial for understanding their stability and durability in real-world applications. Furthermore, the investigation was limited to specific current densities, anode–cathode distances, and electrolyte compositions, leaving the potential for optimization with a broader range of parameters. Lastly, while the findings are significant for battery thermal management systems, the applicability of these composites to other fields, such as heat exchangers or thermal storage systems, remains unexplored and warrants further investigation.

## Figures and Tables

**Table 1:** Parameters applied during the electrolytic Ni-P and Ni-P-Cu coating of PCM/EG composite packs were prepared by pressing and subsequent PCM impregnation.

Sample Code	Coating Type	Distance Between Anode and Cathode (cm)	Current Density (A/dm^2^)
Ni-P1	Ni-P	2	1
Ni-P2	Ni-P	4	2
Ni-P3	Ni-P	6	3
Ni-P-Cu1	Ni-P-Cu	2	1
Ni-P-Cu2	Ni-P-Cu	4	2
Ni-P-Cu3	Ni-P-Cu	6	3

**Table 2:** Thermal data of PCM and PCM/EG composites determined by DSC analysis.

Sample	Melting Onset (°C)	Melting Temperature (°C)	Crystallization Onset (°C)	Crystallization Temperature (°C)	Melting Enthalpy (J/g)	Crystallization Enthalpy (J/g)
Pure PCM	36.79	42.72	42.66	38.32	162	164
EG/PCM	36.23	42.55	43.22	38.33	140	140

## Data Availability

Data will be available on request.

## Supplementary Materials

The following supporting information can be downloaded at: https://www.mdpi.com/article/10.3390/ma18010213/s1, Figure S1: EDS mapping and spectrum images of the Ni-P and Ni-P-Cu coatings obtained with different coating parameters; Table S1: Elemental composition of the coatings obtained by different coating parameters.

## Nomenclature

The following abbreviations and units are used in this manuscript:

PCM	Phase Change Material
EG	Expanded Graphite
Ni-P	Nickel-Phosphorus
Ni-P-Cu	Nickel-Phosphorus-Copper
LIB	Lithium-Ion Battery
DSC	Differential Scanning Calorimetry
TGA	Thermogravimetric Analysis
XRD	X-ray Diffraction
SEM	Scanning Electron Microscopy
EDS	Energy-Dispersive Spectroscopy
RT42	Rubitherm RT42 (specific PCM type)
FDM	Fused Deposition Modeling (3D printing technique for PCM composites)
1.25C	Battery discharge rate at 1.25 times its nominal capacity per hour
2.5C	Battery discharge rate at 2.5 times its nominal capacity per hour
V	Potential (voltage)
A	Current (amperes)
s	Time (seconds)
Wh	Energy capacity (watt-hours)
Ah	Capacity (ampere-hours)
T_m_	Melting temperature
ΔH_m_	Enthalpy of melting
T_c_	Crystallization temperature
ΔH_c_	Enthalpy of crystallization
F_max_	Maximum tensile or compressive stress
k	Thermal conductivity
d_50_	Median particle size
d_10_	Particle size below which 10% of the sample lies
d_90_	Particle size below which 90% of the sample lies
