# Supplementary material for: Electrolytic Ni-P and Ni-P-Cu Coatings on PCM-Loaded Expanded Graphite for Enhanced Battery Thermal Management with Mechanical Properties
Source: Materials (Basel). 2025 Jan 6;18(1):213. doi: 10.3390/ma18010213 (PMC11720822; doi:10.3390/ma18010213)
Supplement: Supplementary file 1 [file materials-18-00213-s001.zip › materials-3385434-supplementary.pdf]

Electrolytic Ni-P and Ni-P-Cu Coatings on PCM-loaded Expanded Graphite for Enhanced Battery Thermal Management with Mechanical Properties

[1]Karadeniz Technical University, Metallurgical and Materials Engineering, Trabzon, TURKEY; onurguler@ktu.edu.tr
[2]Samsun University, Mechanical Engineering, Samsun, TURKEY; myusuf.yazici@samsun.edu.tr
*Correspondence: onurguler@ktu.edu.tr

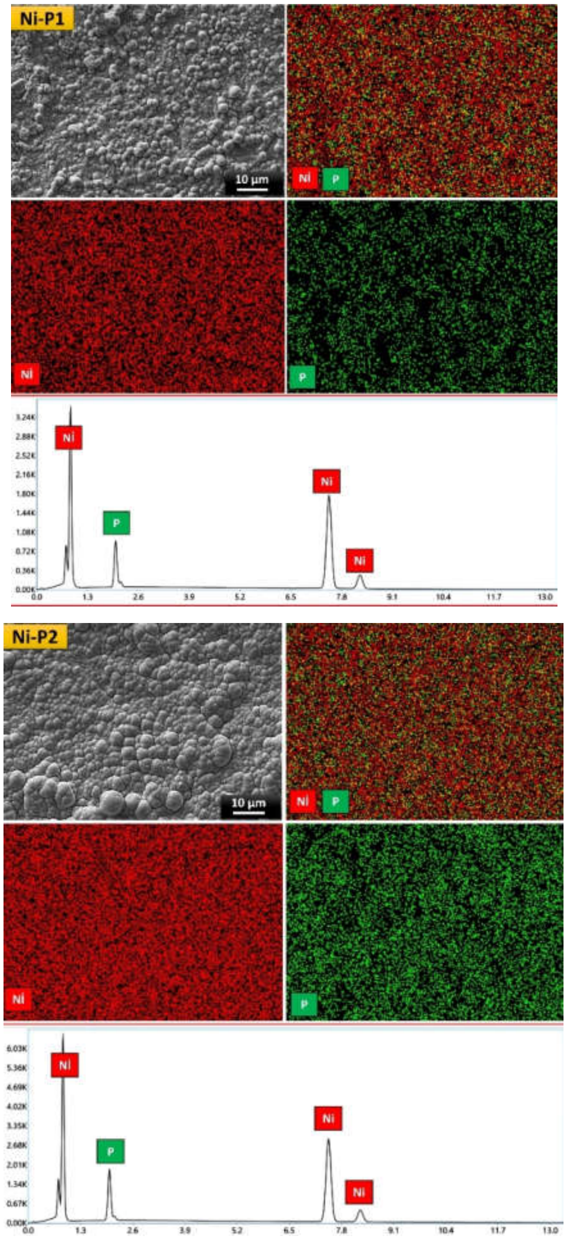

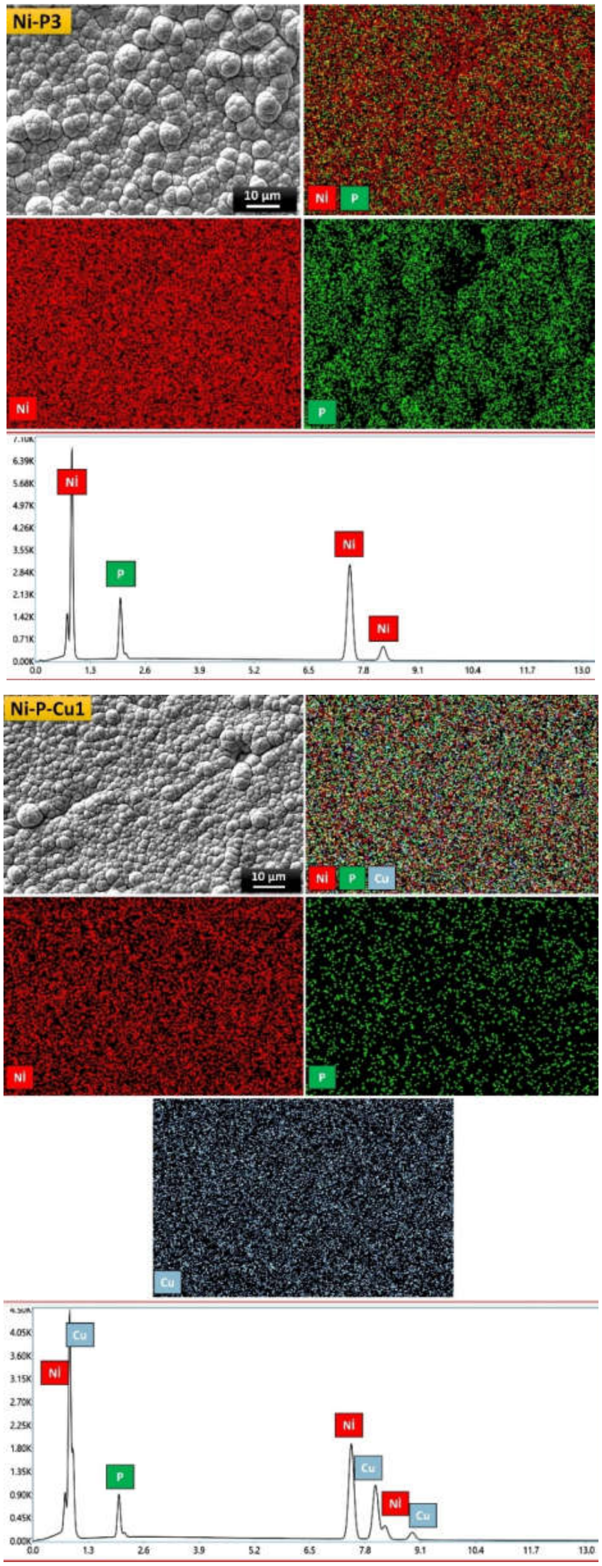

Ni-P3
10 µm
Ni
P
Ni
P
6.39K
5.68K
4.97K
4.26K
3.55K
2.84K
2.13K
1.42K
0.71K
0.00K
0.0
1.3
2.6
3.9
5.2
6.5
7.8
9.1
10.4
11.7
13.0
Ni
P
Ni
Ni
Ni-P-Cu1
10 µm
Ni
P
Cu
Ni
P
Cu
4.05K
3.60K
3.15K
2.70K
2.25K
1.80K
1.35K
0.90K
0.45K
0.00K
0.0
1.3
2.6
3.9
5.2
6.5
7.8
9.1
10.4
11.7
13.0
Cu
Ni
P
Ni
Cu
Ni
Cu

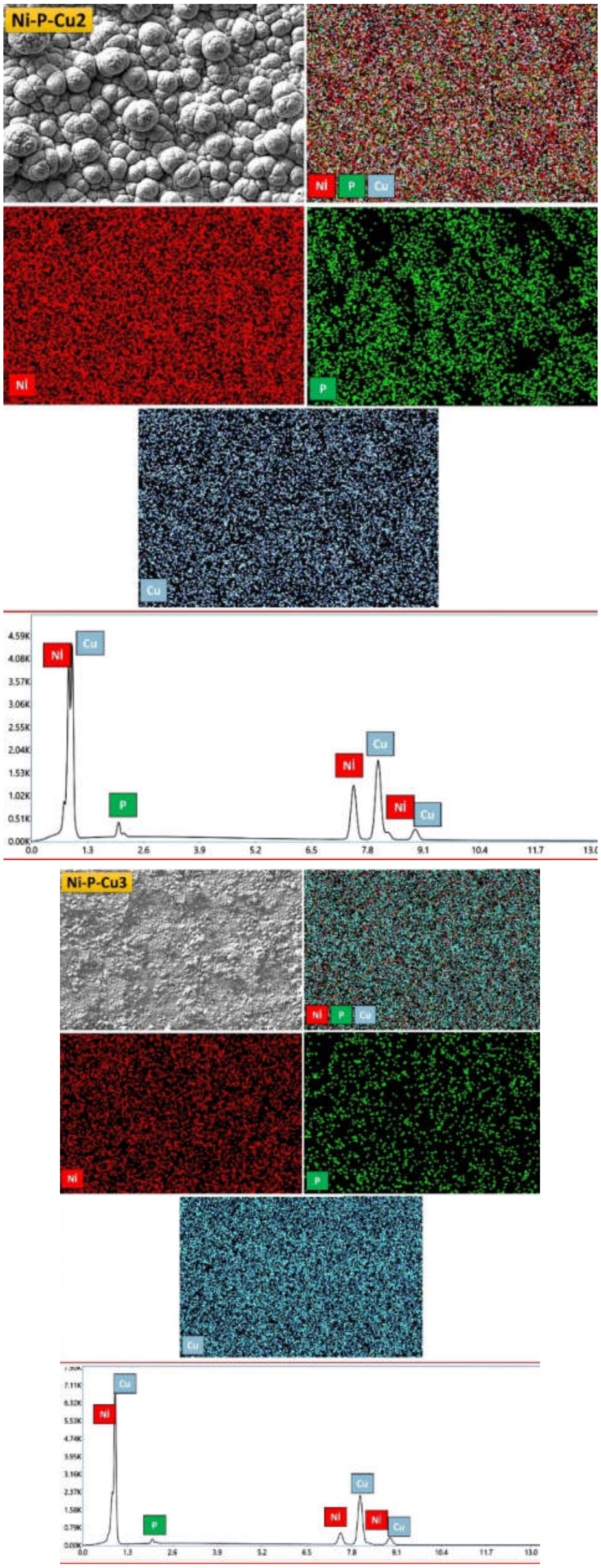

Figure S1. EDS mapping and spectrum images of the Ni-P and Ni-P-Cu coatings obtained with different coating parameters

Table S1. Elemental composition of the coatings obtained by different coating parameters

Element	Ni-P1	Ni-P2	Ni-P3	Ni-P-Cu1	Ni-P-Cu2	Ni-P-Cu3
Ni	88.55	86.30	85.90	61.12	55.47	47.38
P	11.45	13.70	14.10	5.19	3.80	2.50
Cu	-	-	-	33.69	40.73	50.12
